# A Historical Collection of Termites in Ferrara: Recovery, Cataloguing and Geographical Analyses

**DOI:** 10.3390/insects12090793

**Published:** 2021-09-04

**Authors:** Davide Curci, Chiara Scapoli, Maria Gabriella Marchetti, Milvia Chicca, Marilena Leis, Chiara Beatrice Vicentini, Teresa Bonacci, Marco Pezzi

**Affiliations:** 1 Department of Life Sciences and Biotechnology, University of Ferrara, Via L. Borsari 46, 44121 Ferrara, Italy; davide.curci@edu.unife.it (D.C.); chiara.scapoli@unife.it (C.S.); gabriella.marchetti@unife.it (M.G.M.); milvia.chicca@unife.it (M.C.); marilena.leis@unife.it (M.L.); chiara.vicentini@unife.it (C.B.V.); 2 Department of Biology, Ecology and Earth Sciences, University of Calabria, Via P. Bucci, Arcavacata di Rende, 87036 Cosenza, Italy; teresa.bonacci@unical.it

**Keywords:** caste, cataloguing, distribution, foreign, Italian, museum collection, termites

## Abstract

**Simple Summary:**

Termites, an insect group relevant for recycling of organic matter, are also biodeteriogenic organisms that cause serious damages to wooden structures in anthropogenic environments. Professor Antonio Springhetti, a renowned Italian entomologist who studied termites in Italy from 1950 to around 1990, collected many specimens during his field campaigns, and his collection was enriched by termite samples from around the world donated by other entomologists. His precious collection, preserved at the University of Ferrara, represents not only a valuable scientific tool for studies on ecologically relevant insects that may seriously damage historical buildings and ancient libraries but also an important cultural asset.

**Abstract:**

Termites are an insect group relevant for recycling of organic matter, but they are also biodeteriogenic and may cause serious damages to wooden structures (including historical buildings and ancient libraries) in anthropogenic environments. The collection of Italian and foreign termites gathered over the years by Antonio Springhetti, Professor of Zoology at the University of Ferrara (Ferrara, Italy) and internationally renowned entomologist, contains over 44,000 specimens, collected by Springhetti during his field campaigns or donated by other entomologists from all over the world. The collection is currently preserved at the Department of Life Sciences and Biotechnology of the University of Ferrara. Unfortunately, all documents, publications and notes concerning the Springhetti Collection were lost; thus, in 2020, the collection was completely re-catalogued within the University Museum System and analyzed in detail. The collection contains specimens dating back to 1878 and represents not only a valuable scientific tool for studies on these ecologically relevant insects that may cause damages to historical buildings, ancient books and artworks but also an important cultural asset for the University Museum System.

## 1. Introduction

Antonio Springhetti (1923–1992), a renowned Italian entomologist and naturalist, was a pioneer in the study of Italian and foreign termites together with his distinguished mentor, Professor Carlo Jucci, with whom he graduated in Natural Sciences in Pavia (Italy). In 1966, Springhetti arrived at the University of Ferrara (Italy), where he became Full Professor of Zoology in 1984. His studies mostly concerned the social control of caste differentiation in *Kalotermes flavicollis* (Fabricius) (Isoptera: Kalotermitidae), and his contributions to the knowledge of mechanisms of social and endocrine control of this differentiation were universally recognized as fundamental. In addition to laboratory research, the academic activity of Professor Springhetti was characterized by numerous and prolonged field campaigns, where he personally collected termite specimens in almost all the Italian territory, including the islands [1]. The result of these campaigns is a precious and historical collection of ethanol-preserved termite specimens, documenting the Italian populations of *K. flavicollis* and of *Reticulitermes lucifugus* Rossi (Isoptera: Rhinotermitidae) from 1950 to around 1990, as well as specimens (under investigation) that could belong to other species of the genus *Reticulitermes*. Over the years, Professor Springhetti enriched the collection with numerous and precious specimens of termites from all over the world, donated by scholars and entomologists with whom Springhetti had maintained a close correspondence during his academic activity.

Professor Springhetti’s collection of Italian and foreign termites (hereafter indicated as the Springhetti Collection), currently maintained at the Department of Life Sciences and Biotechnology of the University of Ferrara, within the University Museum System (SMA), had a troubled history. After the death of Professor Springhetti in 1992, the collection was preserved and studied by Giovanni Sbrenna, renowned entomologist and Professor of Zoology at the University of Ferrara from 1992 to 2011. However, these studies were interrupted due to the premature academic retirement of Professor Sbrenna, who sadly passed away in 2013. The collection was then relocated many times in the premises of the department, with consequent neglect and loss of original documents, publications and notes by Professor Springhetti.

## 2. Materials and Methods

**Figure 1:**
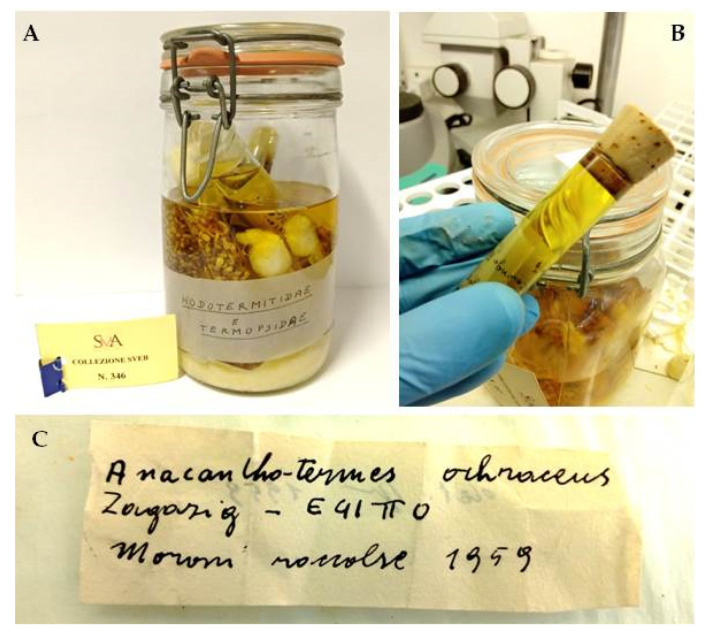
Springhetti Collection. (**A**) Example of a Korken glass jar (jar 18) containing the termite specimens, with its original label and the assigned SMA cataloguing number. (**B**) Example of a test tube with specimens preserved in alcohol. (**C**) Example of an original label with general information about the specimens.

A detailed museographic, biological and geographic research was carried out on the Springhetti Collection. The collection currently includes 40 Korken glass jars with lids, containing Italian and foreign termites preserved in ethanol solution inside glass test tubes, in turn immersed in ethanol. In order to re-catalogue and analyze the collection, all jars were gathered in a safe place for conservation and prepared for cataloguing within the University Museum System (SMA). An identification tag with an SMA catalogue number was assigned to each jar (Figure 1A). Each jar was opened, and all test tubes were extracted and carefully emptied (Figure 1B). All termite specimens contained in each test tube were divided by caste, counted and documented by photographs. Any original label found in the test tube was also recorded and documented by photographs, including labels with initials, probably referring to a personal register kept by Professor Springhetti that is now lost. The specimens were then inserted again in the same test tubes, accompanied by their original label and by a new label with a code indicating the original number of the jar and the test tube number. When the original ethanol solution was partially evaporated, a new 85% ethanol solution was added. Any test tube found damaged was replaced by a new one of the same size, with a cork or a cotton plug similar to the original one. For all jars, the original gaskets, worn out by time, were replaced with new gaskets. All data indicated on the original labels (Figure 1C) (species, date of collection, place of collection, name of the collector and who determined the species, when available) were recorded on an Excel file. The determination of species was based on indications by Professor Springhetti and other scholars, properly verified by the use of dichotomous keys and available literature [2,3,4]. Figures and tables were prepared to describe the data of the collection and facilitate their consultation. Tables S1–S31 were inserted in the Supplementary Material.

For detailed observations under the stereomicroscope, samples chosen from the collection were temporarily stored in 1.5 mL Eppendorf tubes in 85% alcohol, with a label indicating the reference code of the test tube and the jar from which the specimens were taken. Observations were conducted under a Nikon SMZ 800 stereomicroscope (Nikon Instruments Europe, Amsterdam, The Netherlands) connected to a Nikon Digital Sight Ds-Fil camera (Nikon Instruments Europe).

The maps used in the geographic part of the study were drawn by the open source software QGIS [5]. Thanks to this open source program, it is possible to create geographical maps and insert vector points corresponding to locations. The outlines of the Italian Regions were directly downloaded from the official website of the “Istituto Nazionale di Statistica” (Italian National Institute of Statistics, ISTAT) [6]. The points on the maps represented the municipalities where the specimens were collected. Specimens derived from laboratory colonies established from individuals collected in the wild were indicated with a different symbol. Maps were also drawn to visualize the collection sites and the total number of individuals divided by geographic area.

## 3. Results and Discussion

Professor Springhetti’s collection of termite specimens (hereafter Springhetti Collection) is composed of 40 Korken glass jars with lids, containing Italian and foreign termites. Each jar had an original label on the outside bearing an Arabic or Roman number, except for one labelled “Alfieri”. There are 14 jars containing Italian termites, and their specimens come from almost all the Italian territory, while there are 17 jars containing foreign termites, and their specimens come from all continents except Antarctica. The remaining nine jars (15, 39, 48, 49, I, II, III, IV, and “Alfieri”) were found empty or contained specimens without identification, locality of collection or any other information.

Concerning the number of specimens, the collection is presently composed of over 44,000 individuals of various castes, of which about 15,000 are from Italian locations and about 29,000 from foreign countries. During the re-cataloguing, the original numbering and naming of the jars were preserved, but unfortunately, some numbers in sequence (and presumably the corresponding jars) were missing. The jars that did not have any label were numbered. The collection was then catalogued and included in the SMA (Figure 1A).

The specimens of the collection were originally preserved in ethanol and, as previously mentioned, contained in glass test tubes. The number and size of test tubes in each jar were variable, and the test tubes were sealed with cotton plugs, cork or plastic stoppers.

Each tube contained, in addition to the specimens, a label with general information about the content (Figure 1C): taxonomic data (genus, species and/or family), place of origin, date of collection, information about the person who collected the sample (the “legit”) and other observations. The specimens of Italian termites were collected mainly by Professor Springhetti during his field campaigns in Italian Regions, while those of foreign termites were collected by researchers and entomologists from different parts of the world.

Tables S1–S31, reporting in detail the content of each jar, are included in the Supplementary Material. Tables S1–S14 report for each jar the test tube code, the taxa (when indicated), the number of individuals for each caste, the year of collection, the administrative region, the municipality and/or locality of collection for the Italian termites *Kalotermes flavicollis* (Fabricius) (Isoptera: Kalotermitidae) and *Reticulitermes lucifugus* Rossi (Isoptera: Rhinotermitidae). Tables S15–S31 report for each jar the same data about foreign termites collected from all continents. The data reported in the original labels with a question mark were reported as such in the Tables.

### 3.1. Italian Termites

Jar 1 is the only one containing 415 specimens exclusively from laboratory colonies, presumably derived from individuals collected in the wild. These specimens cannot be considered for geographic distribution, as they lack provenance. The biological cycle of termites is altered in laboratory conditions, and castes unlikely to originate in the wild may appear in laboratory colonies due to environmental and/or social factors. Actually, this is the only jar containing *R. lucifugus* ergatoids, i.e., wingless neotenic reproducers directly derived from workers. The number of ergatoids in jar 1 is 23.

Jar 2 contains a total of 2350 specimens, collected from 1954 to 1988, of which 2214 are *R. lucifugus* and 136 are *K. flavicollis*. In order of abundance of individuals, the administrative regions represented for *R. lucifugus* are Lombardy, Emilia-Romagna, Tuscany, Lazio, Umbria; Liguria, Marche and Friuli Venezia-Giulia, and for *K. flavicollis* Emilia-Romagna and Liguria. According to recent classifications [7], termites of the genus *Kalotermes* may belong to the subspecies *K. flavicollis* “sensu strictu”, widespread from the Aegean Sea to Italy. Termites of the genus *Reticulitermes* may belong to the subspecies *R. lucifugus lucifugus*, widespread in the majority of Italian territory, but specimens from Tuscany may belong to the subspecies *R. lucifugus corsicus*, widespread in Sardinia, Corsica, and Tuscany. There is only one specimen labelled “laboratory”, from Bagnacavallo (Ravenna). Here, another species very similar to *R. lucifugus*, *Reticulitermes urbis* Bagnères, Uva & Clément (Isoptera: Rhinotermitidae), has been reported [8,9,10]; thus, it cannot be excluded that some *R. lucifugus* specimens in the collection may actually be *R. urbis*.

In 1958, Springhetti published a study on termites in the Region Marche [11], a location for which very few reports existed at that time. In 1959, he also published another study concerning the first finding of termites in Lombardy [12] in which he analyzed the case of a building infested by termites in the town of Lodi. The building was a two-storey cheese warehouse where the termites had established colonies on the wooden shelves storing the maturing Parmigiano-Reggiano cheese wheels. During the inspection, Springhetti found traces of wood dust and walkways and, after moving one of the cheese wheels, collected termite individuals that he later identified as *R. lucifugus*. The high percentage of humidity in the room and the ceiling composed of wooden beams favoured the proliferation of the termite colony, which would have been difficult on the outside because of cold winters in Lombardy. These studies show the ability of *R. lucifugus* to spread in anthropic environments when conditions are favourable for its life cycle. In 1964 Springhetti studied an infestation of *R. lucifugus* in Salsomaggiore (Parma) [13], which involved several buildings, including private homes and public places. The hypothesis was that the species had reached the town through the import of infested construction or combustion wood and could proliferate over time because of the favourable conditions inside the buildings. In 1987, Professor Guido Campadelli, an entomologist at the University of Bologna (Bologna, Italy), described an infestation by *R. lucifugus* in Bagnacavallo (Ravenna), the first report on the presence of this species in the sub-region Romagna [14]. Furthermore, in this case, the termite colonies were found inside houses (door frames, jambs and wooden beams) and the workers most probably ascended from tunnels dug into the foundations. Again, the termite invasion was ascribed to the import and local transport of previously infested timber.

Jar 3 contains a total of 1868 specimens of *R. lucifugus* collected from 1953 to 1983. In order of abundance of individuals, the administrative regions represented are Apulia, Basilicata and Calabria. According to recent classifications [7], these termites should belong to the subspecies *R. lucifugus lucifugus*, but it is possible that some specimens are *R. urbis*, reported in the Apulian territory [15]. In 1953, Springhetti, together with Carlo Jucci, investigated Apulia for termite populations, carrying out surveys in vineyards on wooden stakes used for supporting vines, which proved to be an optimal habitat for these insects [16]. In Gallipoli (Lecce), out of 100 vine plants examined, only seven contained colonies of *R. lucifugus*; therefore, the infestation was considered minimal. Concerning the localities from Gallipoli to the southern end of the Salento peninsula, he also reported that the emerging of winged adults and the consequent swarming of *R. lucifugus* could also occur in autumn, as for *K. flavicollis* [16]. In 1965, he published his studies on the presence of termites in Basilicata and the surrounding provinces [17] in which he enlisted the specimens collected in July and September 1964. Among the localities mentioned, there were Matera and two of its municipalities (Bernalda and Garaguso) and Potenza and some of its municipalities (Acerenza, Melfi, Rionero in Vulture, Moliterno, Lagonegro, Lauria, Trecchina, Maratea and Latronico). The same study also mentioned the locality of Foggia. Springhetti considered *R. lucifugus* more widespread than *K. flavicollis* in Basilicata and the bordering provinces: out of 43 municipalities investigated, *R. lucifugus* was found in 32 and *K. flavicollis* in 15. Almost all the captures of *R. lucifugus* occurred at altitudes below 700 m a.s.l. and those of *K. flavicollis* at altitudes below 300 m a.s.l. These data highlight the greater adaptability of *R. lucifugus* to lower temperatures and higher altitude soils.

Jar 4 contains a total of 612 specimens of *R. lucifugus*, collected from 1961 to 1982. In order of abundance of individuals, the administrative regions represented are Campania and Molise. According to recent classifications [7], these specimens should be *R. lucifugus lucifugus*. In 1968, Springhetti published a study on the spread of termites in Campania [18], in which he enlisted the specimens collected in previous years in this territory. Among the sampling localities there were the island of Procida (Naples), the municipalities of Maddaloni (Caserta), Lapio and Ariano Irpino (Avellino), Fisciano and Pertosa Grotte (Salerno).

Jar 5 contains a total of 1355 specimens of *R. lucifugus*, collected from 1963 to 1980. In order of abundance of individuals, the administrative regions represented are Sardinia and Sicily. The termite specimens from Sardinia should be *R. lucifugus corsicus*, while those from Sicily should belong to a Sicilian subspecies [7]. In 1963, Springhetti conducted his ninth exploration of Sicily for termite populations, in which he studied the species and evaluated their relative frequency [19]. In an orchard in the locality Pantano (Ribera, Agrigento), at about 5 km from the sea, out of 18 wooden stakes examined in vineyards, six resulted infested by *R. lucifugus* and one by both *R. lucifugus* and *K. flavicollis*. In Porto Empedocle (Agrigento), in a vineyard in the locality Durrueli at about 6 km from the sea, out of over 40 wooden stakes examined, only two were infested by *R. lucifugus*. Springhetti noted that that the soil was more humid and distant from the sea than in other examined areas. In Licata (Agrigento), in the locality Monserrato about 2 km from the sea, one out of five stakes examined was infested with *R. lucifugus*. In Polizzi Generosa (Palermo), in the locality Molini, three out of 33 hazelnut stumps hosted colonies of *R. lucifugus*. On the island of Santa Maria Salina (Messina), in the town gardens, only 1 out of 13 stakes was infested with *R. lucifugus*. Some specimens were sent to Springhetti by L. Giustinoni, an engineer who captured them near Caltagirone (Catania): these specimens are probably those contained in test tube V5-P12. At the end of the expedition in Sicily, Springhetti concluded that *R. lucifugus* was less frequent near the seacoasts than *K. flavicollis*, the most common termite species in Sicily. Concerning Sardinia, the study by Romolo Prota in 1962 should be mentioned, in which the author described the termite infestations in the island, enlisting the affected localities [20]. In the city centre of Sassari, he reported frequent collapses of roofs and floors due to enormous damages caused by *R. lucifugus*. The methods used at that time to fight *R. lucifugus* infestations were inadequate because they were mostly limited to burning “contaminated” materials. Other Sardinian municipalities mentioned in the study by Prota were Dorgali and Oliena (Nuoro), Carbonia (South Sardinia) and Morgongiori (Oristano).

Jars 6, 7 and 8, respectively, contain a total of 1381, 1465 and 366 specimens of *K. flavicollis* from Sardinia. The specimens were, respectively, collected from 1965 to 1994 (jar 6), from 1964 to 1975 (jar 7) and from 1966 to 1976 (jar 8). Concerning jar 6, some specimens were probably added in 1994 by Professor Sbrenna, who cared for the collection after the death of Professor Springhetti in 1992. Jar 7 also contains about a hundred eggs of *K. flavicollis*. The specimens from Sardinia should belong to the Sardinian and Corsican subspecies of *K. flavicollis*, but those found in the southwestern Sardinian territory may belong to *K. flavicollis* “sensu strictu” [7]. In his 1962 study, Prota reported several municipalities of the province South Sardinia as affected by termite infestations, such as Sant’Antioco and Domusnovas, and the province of Oristano. Prota observed that, with some exceptions, all infestations of *K. flavicollis* involved live trees belonging to the arboreal heritage of 33 municipalities. Prota concluded that the species preferred live plants and considered it a primary phytophagous, unlike *R. lucifugus*, which preferred dead wood, such as stumps or wooden house structures.

Jar 9 and 10, respectively, contain a total of 665 and 1351 specimens of *K. flavicollis* from Sicily, respectively collected from 1963 to 1978 and in 1965. These specimens should belong to *K. flavicollis* “sensu strictu” [7]. As previously mentioned for *R. lucifugus*, during the ninth exploration of Sicily in 1963 [19], in an orchard in the locality Pantano (Ribeira, Agrigento) about 5 km from the sea, in 11 out of 18 wooden stakes examined, Springhetti found infestations by *K. flavicollis* and in one by both *K. flavicollis* and *R. lucifugus*. In the same area, four out of six necrotic olive trees hosted colonies of *K. flavicollis* and in the locality Monserrato of Licata, about 2 km from the sea, one out of five stakes examined was infested with *K. flavicollis*. In the locality Caduta Sul Mare, two stakes out of four hosted this species. Near Palma di Montechiaro, about 15 km from the town, 3 out of 14 stakes examined were infested with *K. flavicollis*. In the locality of Siculiana, about 2 km from the sea, colonies of *K. flavicollis* were found in the necrotic parts of carob trees. At the end of the expedition, Springhetti concluded that *K. flavicollis* was frequent in Sicily, both near the coasts and on inland.

Jar 11 contains a total of 981 specimens of *K. flavicollis* from Apulia, collected from 1953 to 1976. These specimens should belong to “*K. flavicollis* sensu strictu” [7]. During his exploration of Apulia in 1953 [16] in a vineyard in Gallipoli (Lecce), Springhetti found 40 out of 100 vines (40%) infested by *K. flavicollis*, thus a high degree of infestation. In this jar, the test tube V11-P50, which referred to Gallipoli, contains the highest number of pseudergates (304 on a total of 319 specimens) in comparison to all other test tubes containing *K. flavicollis* specimens, suggesting that the colonies observed in this location were old and with a high number of individuals.

Jar 12 contains a total of 227 specimens of *K. flavicollis*, collected from 1964 to 1980. In order of abundance of individuals, the administrative regions represented are Calabria and Basilicata. The specimens should belong to “*K. flavicollis* sensu strictu” [7]. In his study on termites in Basilicata and neighbouring provinces [17], Springhetti enlisted among the localities where he collected *K. flavicollis* Montalbano Jonico (Matera) on necrotic parts of olive and citrus plants, Bernalda (Matera) on wooden stakes, and Maratea (Potenza) on live plants. Springhetti considered *K. flavicollis* less frequent than *R. lucifugus* in Basilicata and neighbouring provinces. Almost all captures of *K. flavicollis* in 1964 were made below 300 m a.s.l., probably the limit beyond which this species cannot adapt to lower temperatures. In contrast, captures of *R. lucifugus* occurred up to 700 m a.s.l., showing that this species can withstand lower temperatures. This result is relevant to explain the different geographic distribution of the two species. In Basilicata, however, there was a narrow location that Springhetti considered immune to the presence of termites, that is, the mountain area of Lucania Apennines, characterized by cold winters with frequent snowfall [17].

Jar 13 contains a total of 356 specimens of *K. flavicollis* from Campania, collected from 1961 to 1981. According to recent classifications [7], the specimens should belong to “*K. flavicollis* sensu strictu”. In his study on diffusion of termites in Campania [18], Springhetti enlisted among the localities where he collected *K. flavicollis* Lapio (Avellino), Capua and Maddaloni (Caserta), Portici and Procida (Naples), Fisciano, Pertosa Grotte and Sarno (Salerno), and Telese Terme (Benevento). The wooden structures that housed the termites were mostly plants of various species and wooden stakes to support the vines. Springhetti also reported to have found winged individuals with dark pronotum at Vairano, Capua and Maddaloni (Caserta).

Jar 14 contains a total of 1495 specimens of *K. flavicollis*, collected from 1958 to 1991. In order of abundance of individuals, the administrative regions represented are Marche, Abruzzo, Tuscany, Umbria and Emilia-Romagna. The specimens should belong to “*K. flavicollis* sensu strictu” [7] but those from Tuscany could belong to the Sardinian-Corsican subspecies. In jar 14, there are also termites from Marina Alberese (Grosseto), where a new species, *Kalotermes italicus* sp. nov. Ghesini & Marini was recently reported [21]. In his study on the exploration of the Region Marche [11], Springhetti enlisted Ancona, Mondolfo (Pesaro-Urbino), Grottammare (Ascoli Piceno), and Macerata as localities where he collected *K. flavicollis*, but no specimens from these localities were present in the collection.

Jar 15 does not contain any specimen: we may advance the hypothesis that the specimens originally contained in the jar had been sorted in other jars, or that they were used for studies that resulted in their destruction.

**Figure 2:**
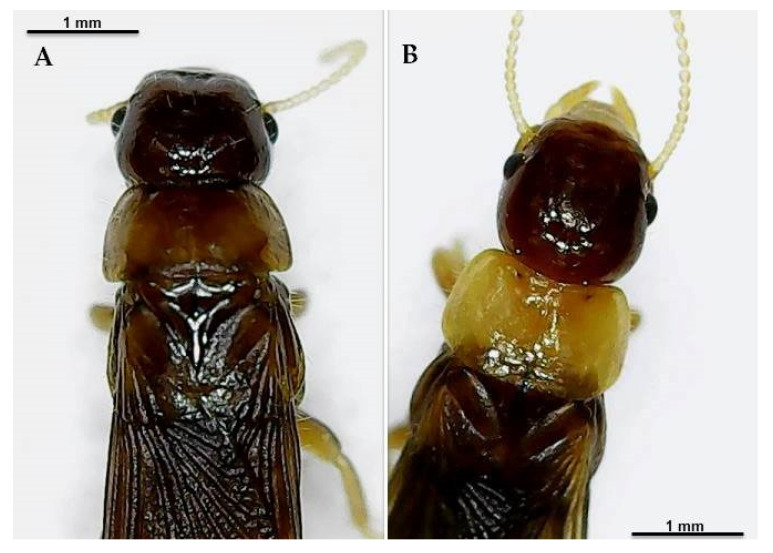
Alate individuals of *Kalotermes* sp. (**A**) Alate probably belonging to *Kalotermes italicus* sp. nov. contained in jar II. (**B**) Alate of *Kalotermes flavicollis* contained in jar 6, tube V6-P06.7.

Concerning jars 39, 48, 49, the jars with Roman numerals I, II, III, IV, and the jar called “Alfieri”, we may advance the hypothesis that they contain reference specimens for identification of other specimens. Jar I, II, IV and “Alfieri” may contain specimens of great interest, (Figure 2), probably belonging to the recently described new species *K. italicus* sp. nov. [21].

### 3.2. Foreign Termites

Jar 17 contains 921 specimens of *Mastotermes darwiniensis* Froggatt (Isoptera: Mastotermitidae) from Australia, collected from 1913 to 1959. This is the only species in the genus *Mastotermes* Froggatt that is native to Australia, and it is the oldest surviving species of termites [22].

Jar 18 contains a total of 1450 specimens of various termite species, collected from 1958 to 1981 in Africa, except those contained in test tube V18-P03, collected in California (North America). About half of the test tubes contain specimens of *Anacanthotermes ochraceus* (Burmeister) (Isoptera: Hodotermitidae). Concerning the specimens from North America, we may advance the hypothesis that they belong to the genus *Zootermopsis* Emerson, presumably *Zootermopsis angusticollis* Hagen (Isoptera: Archotermopsidae) [22].

**Figure 3:**
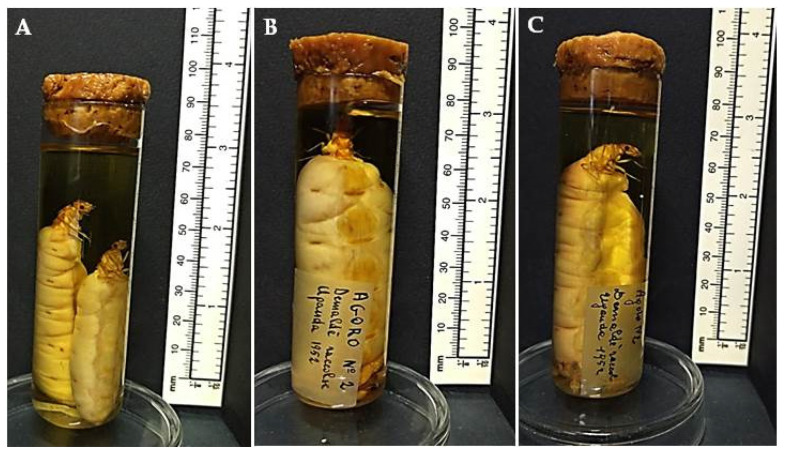
Examples of physogastric queens from Africa, respectively, contained in tubes V19-P03 (**A**), V19-P06 (**B**) and V19-P08 (**C**).

Jar 19 contains a total of 100 specimens, all from Africa except those in test tube V19-P11, from Pakistan. All specimens were collected from 1952 to 1953. This jar also contains physogastric queens from Africa (Figure 3). Unfortunately, the queens were not determined, but we may advance the hypothesis that they belong to the genus *Macrotermes* Holmgren (Isoptera: Termitidae) [22]. Queens of this genus are known to reach very large sizes, up to 10 cm in length. The collection locality is indicated on labels as “Oubangui-Chari”, a French territory in Central Africa that became the independent state of the Central African Republic in 1960. The specimens from this territory were collected by De Genes in 1953, thus before the establishment of the new state.

Jar 20 contains a total of 1103 specimens from all continents, collected from 1878 to 1971. In this jar, there are the oldest specimens of the collection, six alate individuals of *Stolotermes brunneicornis* (Hagen) (Isoptera: Stolotermitidae), collected in 1878 in Titirangi (New Zealand). Some of the species contained in jar 20 are indicated with an obsolete nomenclature: *Incisitermes schwarzi* (Banks) (Isoptera: Kalotermidae) is indicated as “*Kalotermes schwarzi*”, *Marginitermes hubbardi* (Banks) (Isoptera: Kalotermitidae) as “*Kalotermes hubbardi*”, *Psammotermes hybostoma* Desneux (Isoptera: Rhinotermitidae) as “*Psammotermes assuanensis*” and *Schedorhinotermes medioobscurus* (Holmgren) (Isoptera: Rhinotermitidae) as “*Schedorhinotermes javanicus*”. Concerning the collection locality, the Democratic Republic of Congo is referred to as “Congo Belga” (Belgian Congo) because the specimens were collected in 1957, before the date of independence of the new state in 1960. Similarly, the Republic of Benin is indicated as “State of Dahomey”.

Jar 21 contains a total of 4013 specimens collected from 1952 to 1964, in order of abundance of individuals from Africa, Central America, Asia and Oceania. For most specimens, no identification is provided, but it is likely that those from Senegal, Somalia, Central African Republic and Uganda belong to the genus *Macrotermes*. Only two species were identified: *Macrotermes subhyalinus* (Rambur) (Isoptera: Termitidae) indicated as “*Bellicositermes jeanneli*?” and *P. hybostoma* indicated as “*Psammotermes assuanensis*”. The Central African Republic is indicated on labels as “Oubangui-Chari”.

Jar 22 contains a total of 3846 specimens collected from 1924 to 1960, in order of abundance of individuals from Africa, Asia and South America. Only four test tubes have a label with a determination. The genus *Nasutitermes* Dudley (Isoptera: Termitidae) is indicated as “*Eutermes*” and the species *S. medioobscurus* as “*Schedorhinotermes javanicus*”.

Jar 23 contains a total of 4186 specimens collected from 1922 to 1955, in order of abundance of individuals from Africa, South America and Asia. Only three test tubes have a label with a determination. The species *Pseudacanthotermes militaris* Hagen (Isoptera: Termitidae) is indicated as “*Pseudacanthotermes minor*?” and *S. medioobscurus* as “*Schedorhinotermes javanicus*”. The Central African Republic is indicated on labels as “Oubangui-Chari”. The State of Tanzania is indicated as “Tanganyika”, an East African state that merged with the island of Zanzibar in 1964, forming the new State of Tanzania.

Jar 24 contains a total of 3316 specimens collected from 1952 to 1971, in order of abundance of individuals from Africa, Asia and Oceania. None of the test tubes has a label with determination except V24-P14. The only species determined is “*Psammotermes fuscofemoralis*”, currently identified as *P. hybostoma*. The State of Sri Lanka is indicated as “Ceylon”, the official name of the state until 1972. The specimens were collected in 1971, the year before the renaming of the state.

Jar 26 contains 136 winged individuals, collected in 1952 in Uganda. None of them were determined, but they probably belong to the genus *Macrotermes*.

Jar 29 contains a total of 1332 specimens collected from 1952 to 1958, in order of abundance of individuals from South America, Asia, Central America and Africa. The only species identified was *Cornitermes cumulans* (Kollar) (Isoptera: Termitidae), native to South America. Probably the specimens from Uganda belong to the genus *Macrotermes*.

Jar 30 contains a total of 1222 specimens collected from 1952 to 1972, in order of abundance of individuals from South America, Africa, Asia, Central America and Oceania. The specimens of the genus *Macrotermes* are indicated as “*Bellicositermes*”: *Macrotermes bellicosus* (Smeathman) is indicated as “*Bellicositermes bellicosus?*”, *Macrotermes falciger* (Gerstäcker) as “*Bellicositermes goliath*?”, *Macrotermes subhyalinus* (Rambur) as “*Bellicositermes jeanneli*?”, *Macrotermes natalensis* (Haviland) is indicated as “*Bellicositermes natalensis*?” and *Macrotermes ukuzii* Fuller is indicated as “*Bellicositermes ukuzii*” (Isoptera: Termitidae). The Central African Republic is indicated on labels as “Oubangui-Chari.” The State of Tanzania is referred to as “Tanganyika”.

Jar 31 contains a total of 2687 specimens collected from 1952 to 1960, in order of abundance of individuals from Africa and Asia. Most specimens in jar 31 were not determined. The specimens from Senegal, Uganda and Zambia probably belong to the genus *Macrotermes*. The State of Zambia is indicated as Northern Rhodesia, a protectorate of the British Empire that ended in 1964. The date found in the test tube is 1952, thus predating the establishment of the State of Zambia.

Jar 32 contains a total of 695 specimens collected from 1922 to 1961, in order of abundance of individuals from Central America, Africa, South America, Europe and Asia. Ten test tubes have no labels with determination. Some species are indicated with an obsolete nomenclature: *M. bellicosus* is indicated as “*Bellicositermes convexus*?”, *Nasutitermes schoutedeni* (Sjöstedt) as “*Eutermes aethiops*” and *Nasutitermes incurvus* (Sjöstedt) (Isoptera: Termitidae) as “*Eutermes incurvus*”. The Central African Republic is indicated as “Oubangui-Chari”. The Democratic Republic of Congo is indicated as “Congo Belga” (Belgian Congo).

Jar 33 contains a total of 1139 specimens collected from 1920 to 1981, in order of abundance of individuals from America, Africa and Asia. Among the test tubes, twelve have no labels with determination. The Democratic Republic of Congo is indicated as “Congo Belga” (Belgian Congo).

Jar 34 contains a total of 1563 specimens collected from 1952 to 1956, in order of abundance of individuals from Africa, Oceania and Asia. None of the specimens bears a label with identification, but those from Central African Republic, Senegal, Sierra Leone and Uganda probably belong to the genus *Macrotermes*.

Jar 37 contains a total of 819 specimens collected from 1930 to 1962, in order of abundance of individuals from Asia, Africa, South America, Oceania, Europe and North America. The species *Hospitalitermes nemorosus* Ghidini (Isoptera: Termitidae) is indicated as “*Lacessitermes nemorosus*”, *M. natalensis* as “*Bellicositermes natalensis*” and *S. medioobscurus* as “*Schedorhinotermes javanicus*”. The Central African Republic is indicated on labels as “Oubangui-Chari”. The Democratic Republic of the Congo is indicated as “Congo Belga” (Belgian Congo), the State of Tanzania as “Tanganyika” and the State of Zambia as “Northern Rhodesia”. Ghana is referred to as “Gold Coast”, a British colony that became the State of Ghana in 1957.

Jar 38 contains a total of 660 specimens collected in 1989, all probably from the African State of Somalia. None of the test tubes has a label with identification, except tube V38-P03, which bears the label “*Cryptotermes*?”.

### 3.3. Geographic Distribution of Italian Termites

Entomological collections composed of specimens collected in the wild are very important for studies on geographic distribution of insects. In the first half of the twentieth century, many studies were published on first sightings and infestations of termites in the Italian territory. A study on the distribution of termites in Italy was published in 2008 by Giovanni Sbrenna and Anna Micciarelli Sbrenna, including a topographic catalogue and geographic information [23]. In this study, the authors used as reference two previous studies by Antonio Springhetti published in Bollettino dell’Istituto di Patologia del Libro (Bulletin of the Institute of Book Pathology) and the termite specimens contained in the Springhetti Collection. It is therefore useful to compare the data in the study by Sbrenna and Micciarelli Sbrenna (2008) with those obtained from the cataloguing and numbering of the specimens in the Springhetti Collection. The geographic distribution of the Italian termites contained in the collection and any differences with the study published in 2008 are reported below in two paragraphs, the first concerning *K. flavicollis* and the second one *R. lucifugus*.

### 3.4. Geographic Distribution of K. flavicollis in Italy

Concerning the collection sites of *K. flavicollis*, Liguria is represented in the Springhetti Collection by two municipalities, Riomaggiore and Sarzana, both in the province of La Spezia. The locality “Manarola” is indicated for Riomaggiore and the locality “Marinella Sarzana” for Sarzana. Riomaggiore was not mentioned in the study of 2008.

In the Springhetti Collection, Emilia-Romagna is represented by five municipalities: Ferrara, Fiscaglia and Masi Torello (Ferrara), Ravenna and Rimini. The locality “Migliarino” is indicated for Fiscaglia, the locality “Pineta San Vitale” for Ravenna and the locality “San Fortunato” for Rimini. Fiscaglia was not mentioned in the study of 2008.

Tuscany is represented by seven municipalities: Grosseto, Carrara (Massa-Carrara), Massarosa and Viareggio (Lucca), Pisa, Volterra (Pisa) and Poggibonsi (Siena). The locality “Marina di Alberese” is indicated for Grosseto, the locality “Avenza” for Carrara and the name “istituto” “ for Viareggio. Carrara was not mentioned in the study of 2008.

Marche is represented by four municipalities: Ancona, Senigallia (Ancona), Grottammare (Ascoli Piceno), Mondolfo (Pesaro-Urbino). All municipalities are also mentioned in the study of 2008.

Umbria is represented by only one town in the province of Terni, Amelia, also mentioned in the study of 2008.

Abruzzo is represented by 8 municipalities: Chieti and province (Bucchianico, Francavilla a mare, Miglianico, Orsogna and Poggiofiorito), Pescara and Montesilvano (Pescara). The indication “laboratory” is reported for Montesilvano. All municipalities are also mentioned in the study of 2008.

Campania is represented by 12 municipalities: Lapio (Avellino), Telese Terme (Benevento), Capua and Maddaloni (Caserta), Ischia, Barano D’Ischia, Forio, Portici and Procida (Napoli), Fisciano, Pertosa and Sarno (Salerno). The locality “Maddaloni superiore” is indicated for Maddaloni, “porto” for Ischia, “Pertosa grotte” for Pertosa and “Isola di Vivara” for Procida. For Portici, six specimens are present in the Springhetti Collection, one labelled “laboratory”, one personally collected by Springhetti and four with no information about “legit”. All municipalities are also mentioned in the study of 2008.

Apulia is represented by 11 municipalities: Manfredonia, Mattinata, Monte Sant’Angelo, San Giovanni Rotondo and Vico Garganico (Foggia), Andrano, Galatone, Gallipoli, Leuca and Squinzano (Lecce), and Manduria (Taranto). The localities “Massa Mozzillo”, “Massa Sant’Angelo”, “Posta di Scarpetta”, “S. Maria vecchia”, “S. Oronzo” and “Villa Rosa” are indicated for Manfredonia. The localities “Le Monache”, “Massa Liberatore”, “Massa Papone” and “Villa Berenice” are indicated for Mattinata and the locality “Madonna delle Grazie” for Monte Sant’Angelo. The localities “Campolato”, “Cava di pietra”, “Piano Mezzanella” and “Posta Padovano” are indicated for San Giovanni Rotondo. The labels “insectarium” and “various nests” are indicated for Squinzano. All municipalities are also mentioned in the study of 2008.

Basilicata is represented by four municipalities, three from the province of Matera (Bernalda, Montalbano Jonico and Oliveto Lucano), and Maratea (Potenza). All municipalities are also mentioned in the study of 2008.

Calabria is represented by four municipalities: Paola (Cosenza), Belvedere di Spinello (Crotone), Bagnara Calabra (Reggio Calabria) and Spilinga (Vibo Valentia). The locality “Pellegrina” is indicated for Bagnara Calabra. All municipalities are also mentioned in the study of 2008.

Sicily is represented by 16 municipalities: Agrigento and Favara, Lampedusa, Licata, Linosa, Palma di Montechiaro, Ribera and Siculiana (Agrigento), Messina, Palermo, Bagheria and Polizzi Generosa (Palermo), Siracusa, Rosolini (Siracusa), Castelvetrano and Pantelleria (Trapani). The locality “Villa Palagonia” is indicated for Bagheria, the localities “Caduta” and “Monserra” for Licata and the locality “Torretta Giallonghi” for Castelvetrano. All municipalities are also mentioned in the study of 2008.

Sardinia is represented by 21 municipalities: Cagliari, Pula, Quartu Sant’Elena and Sinnai (Cagliari), Ulassai (Nuoro), Oristano, Bonarcado and Santu Lussurgiu (Oristano), Sennori (Sassari) and Barumini, Burcei, Carbonia, Carloforte, Domusnovas, Gonnesa, Las Plassas, Portoscuso, San Giovanni Suergiu, Sant’Antioco, Teulada and Villasimius, all in South Sardinia. The locality “spiaggia di Is Mortorius” (Is Mortorius beach) is indicated for Quartu Sant’Elena and the locality “Campu Omu” is indicated for Sinnai. Quartu Sant’Elena, Santu Lussurgiu and Sinnai are not mentioned in the study of 2008.

Concerning geographic distribution, *K. flavicollis* is almost absent in Northern Italy: Liguria and Emilia-Romagna are the only regions of Northern Italy documented in the Springhetti Collection. The colder climate of these areas is clearly unfavourable for the survival of this species [12,24]. Specimens from regions in central Italy are present in the collection, except for Lazio and Molise. The species is well distributed in Southern Italy and the islands: Sicily and Sardinia are the richest regions for the number of collecting sites. Among municipalities, there are numerous seaside resorts or localities near the sea where specimens of *K. flavicollis* were collected. The species, therefore, exhibits a higher adaptability to the seaside biotope in comparison to *R. lucifugus*. The study of 2008 also indicates Lombardy, Friuli-Venezia Giulia and Veneto as regions where *K. flavicollis* could be found, but in the Springhetti Collection, there are no specimens documented for these regions. However, we cannot exclude the hypothesis that the missing jars contained specimens from these regions.

**Figure 4:**
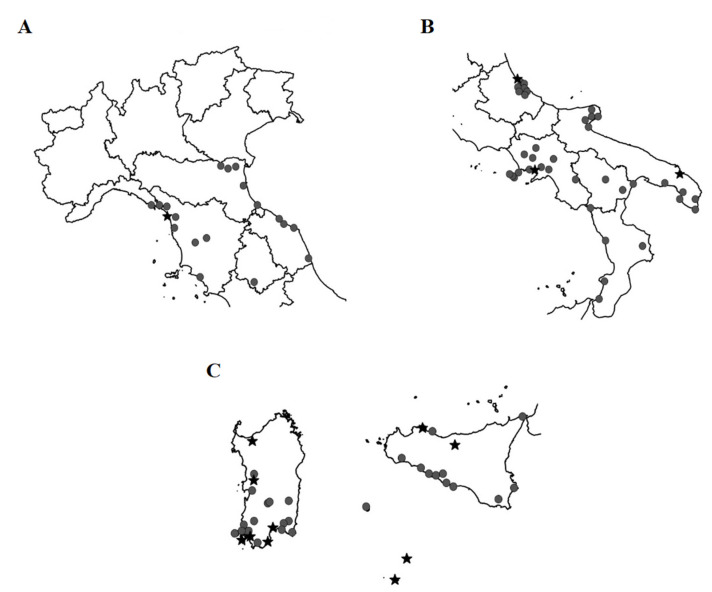
Italian localities where specimens of *K. flavicollis* were collected. (**A**) Northern Italy. (**B**) Southern Italy. (**C**) Sardinia, Sicily and minor islands. Circles, specimens collected in the wild. Stars, laboratory specimens presumably derived from individuals collected in the wild.

The maps showing the localities where *K. flavicollis* specimens were collected are shown in Figure 4A–C, respectively, for Northern Italy, Southern Italy and the Italian islands.

### 3.5. Geographic Distribution of R. lucifugus in Italy

Concerning the collection sites of *R. lucifugus*, Lombardy is represented in the Springhetti Collection by two municipalities, Lodi and Pavia. Pavia is enlisted as “Institute”, probably referring to the Institute of Zoology of University of Pavia. Lodi and Pavia are also mentioned in the study of 2008.

Friuli-Venezia Giulia is represented by only one municipality, Udine, with the locality “Giardino Cardi”. Udine is also mentioned in the study of 2008.

Liguria is represented by only one municipality in the province of Genoa, Camogli, which is not mentioned in the study of 2008.

Emilia-Romagna is represented by two municipalities: Bagnacavallo (Ravenna) and Salsomaggiore Terme (Parma). Three specimens were collected in Bagnacavallo, one specimen labelled “Diff. in laboratorio” (presumably “raised in the laboratory”), one specimen collected by Professor Campadelli, and one without any “legit” or other information. These two municipalities are also mentioned in the study of 2008.

Tuscany is represented by nine municipalities: Livorno, Barberino di Mugello (Florence), Barberino Tavernelle (Florence), Reggello (Florence), Pisa, Bagni di Lucca (Lucca), Camaiore (Lucca), Grosseto and Poggibonsi (Siena). The locality of Barberino Val D’Elsa is indicated for Barberino Tavernelle, San Donato for Reggello, Lido di Camaiore (a seaside resort) for Camaiore and the resort of Pineta del Tombolo for Grosseto. Barberino Tavernelle is not mentioned in the study of 2008.

Marche is represented by only one municipality in the province of Macerata, Castelraimondo, mentioned also in the study of 2008.

Umbria is represented by only one town in the province of Perugia, Todi, also mentioned in the study of 2008.

Laziois represented by two localities: Rome and Antrodoco (Rieti). Two tubes were labelled “Rome”, one with specimens collected by Ghidini, the other with specimens from the Institute of Book Pathology, probably laboratory specimens. Rome and Antrodoco are also mentioned in the study of 2008.

Molise is represented by only one town in the province of Isernia, Miranda, a locality not mentioned in the study of 2008.

Campania is represented by 10 municipalities: Barano d’Ischia, Ischia, Serrara Fontana and Procida (Naples), Maddaloni (Caserta), Lapio, Ariano Irpino, Monteforte Irpino (Avellino), Fisciano and Pertosa (Salerno). The locality “porto” is indicated for Ischia, the locality “Testaccio” for Barano d’Ischia and “Maddaloni superiore” for Maddaloni. The locality “Cerreto stazione” is indicated for Ariano Irpino, “Monteforte superiore” for Monteforte Irpino and “Pertosa grotte” for Pertosa. All municipalities are also mentioned in the study of 2008.

Apulia is represented by 12 municipalities: Foggia and six other towns of the province (Manfredonia, Mattinata, Monte Sant’Angelo, Rocchetta Sant’Antonio, San Giovanni Rotondo and Peschici), Manduria (Taranto), Gallipoli, Leuca, Galatone and Squinzano (Lecce). The locality “Minonno” is indicated for Manfredonia, and “La Cavola” and “Carmine” for Mattinata. The localities of “Bosco Quarto”, “la costa” (the coast), “macchia libera” (maquis shrubland), “Manganera” and “Massa Sansone” are indicated for Monte Sant’Angelo and the locality “stazione” (railway station) for Rocchetta Sant’Antonio. The locality “Funno” is indicated for San Giovanni Rotondo and the locality “Calenella stazione” (Calenella railway station) for Peschici. Two test tubes were labelled “Squinzano”, one with the word “laboratory” and the other without any description. All municipalities are also mentioned in the study of 2008.

Basilicata is represented by 12 municipalities, ten from the province of Potenza (Acerenza, Lagonegro, Latronico, Lauria, Maratea, Melfi, Moliterno, Rapone, Rionero in Vulture and Trecchina), and two from the province of Matera (Bernalda and Garaguso). The locality “Ponte Episcopa” is indicated for Latronico and the locality “Metaponto Bernalda” for Bernalda. All municipalities are also mentioned in the study of 2008.

Calabria is represented by four municipalities: Praia a Mare and Rende (Cosenza), Belvedere Spinello (Crotone), and Bagnara Calabra (Reggio Calabria). The locality “Pellegrina di Bagnara” is indicated for Bagnara Calabra. All municipalities are also mentioned in the study of 2008.

Sicily is represented by eight municipalities: Polizzi Generosa and Bagheria (Palermo), Licata, Porto Empedocle and Ribera (Agrigento), Pantelleria (Trapani), Caltagirone (Catania) and Salina (Messina). The locality “Monserrato” is indicated for Licata, the locality “Pantano” for Ribera, the locality “Durrueli” for Porto Empedocle and the locality for “Villa Palagonia” for Bagheria. All municipalities are also mentioned in the study of 2008.

Sardinia is represented by eight municipalities: Pula, Quartu Sant’Elena and Sinnai (Cagliari), Dorgali and Oliena (Nuoro), Morgongiori (Oristano), Sennori (Sassari) and Carbonia (South Sardinia). The locality “spiaggia di Is Mortorius” (Is Mortorius beach) is indicated for Quartu Sant’Elena, the locality “Campu Omu” for Sinnai and the locality “Cala Gonone” for Dorgali. Quartu Sant’Elena is not mentioned in the study of 2008, but a locality near this town, Flumini di Quartu, is mentioned.

**Figure 5:**
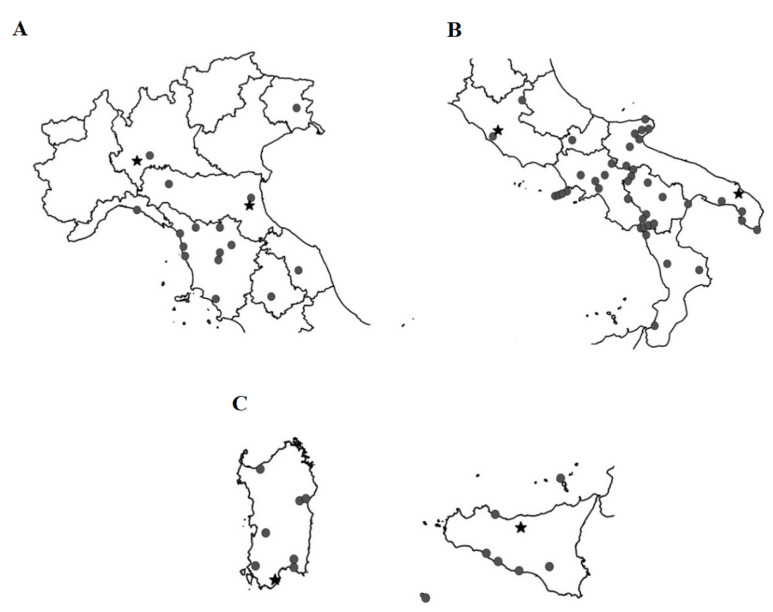
Italian localities where specimens of *R. lucifugus* were collected. (**A**) Northern Italy. (**B**) Southern Italy. (**C**) Sardinia, Sicily and minor islands. Stars, laboratory specimens; Circles, specimens collected in the wild.

In comparison to *K. flavicollis*, *R. lucifugus* is more evenly distributed in the Italian territory (Figure 5A). In Northern Italy, *R. lucifugus* occurs in Lombardy, Friuli-Venezia Giulia [25] and Liguria [23]; thus, this termite species is apparently more resistant to cold climates and altitudes in comparison to *K. flavicollis*. Specimens of *R. lucifugus* are present in all regions of central Italy except Abruzzo. There are no literature reports of captures of *R. lucifugus* in Molise [23], but in the Springhetti Collection, there are specimens collected in Miranda (Isernia). In Southern Italy, the distribution of this species is wider, and Apulia and Campania are the regions with the most numerous sites of collection. In the catalogue of 2008, Abruzzo, Piedmont and Veneto are also indicated [26], but in the Springhetti Collection, there are no specimens of *R. lucifugus* from these regions. Again, we cannot exclude the hypothesis that the missing jars contained specimens from these regions. Interestingly, both subspecies *R. lucifugus lucifugus* and *R. lucifugus corsicus* have been recently signalled in Piedmont [27]. Another allochthonous species of the genus, *Reticulitermes flavipes* (Kollar) (Isoptera: Rhinotermitidae), was recently signalled in Lombardy [28].

The maps showing the localities where *R. lucifugus* specimens were collected are shown in Figure 5A–C, respectively, for Northern Italy, Southern Italy and some Italian islands.

### 3.6. Geographic Distribution of Foreign Termites

**Figure 6:**
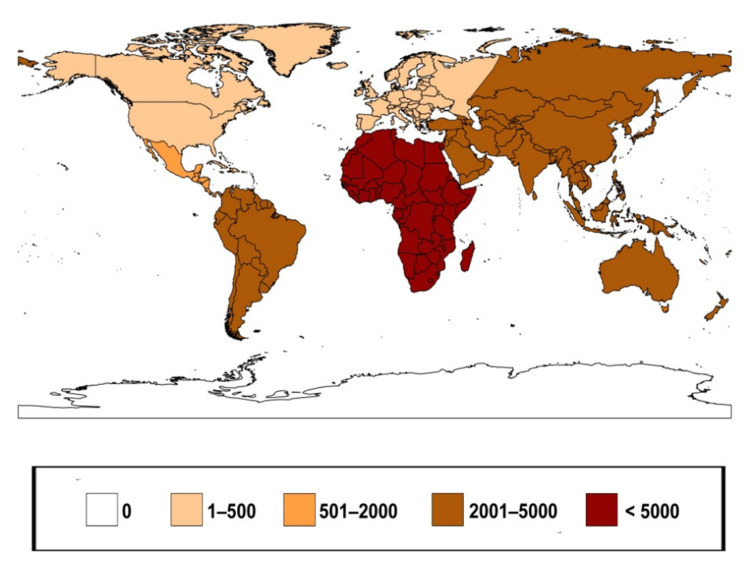
Number of foreign termite specimens in Springhetti Collection according to continent.

The foreign termites contained in the Springhetti Collection amount to 29,188 specimens and represent the main part of the collection. According to the number of specimens, the continents and subcontinents represented are Africa, Asia, South America, Oceania, Central America, North America, and Europe (Figure 6). The African countries represented are Egypt, Senegal, Sierra Leone, Central African Republic, Uganda, Somalia, Kenya, Mozambique, the Democratic Republic of the Congo, Zambia, Tanzania, South Africa, Benin, Ethiopia, Zimbabwe and Ghana. The Asian countries represented are Indonesia (Sumatra), the Philippines, Pakistan, Sri Lanka, Japan, Malaysia, and India. The South American countries represented are Argentina, Brazil, Venezuela and Guyana. The countries of Oceania represented are Papua New Guinea, Australia, and New Zealand. The countries of Central America represented are Panama, Costa Rica and the Bahamas. The North American States represented are California, South Carolina, Arizona, Virginia, Florida, Washington and Texas. The European countries represented (excluding Italy) are Germany, Spain and Portugal.

## 4. Conclusions

The valuable collection of termite specimens from Italy and from all over the world, gathered over the years by Antonio Springhetti, Professor of Zoology at the University of Ferrara and renowned entomologist, was recovered, re-catalogued and safely stored within the University Museum System (SMA), as a precious asset for morphological, molecular and geographic studies. All 40 jars containing the collection were examined and carefully documented. All specimens preserved in ethanol in tubes within the jars were counted and catalogued, except the jars for which it was not possible to obtain reliable information on provenance and year of collection. On all jars, operations aimed at better preservation of the specimens over time were conducted, such as the restoration of adequate ethanol levels to prevent drying of the specimens and the replacement of all gaskets to prevent the evaporation of the ethanol solution.

Concerning the data of the collection, there are 12 Italian regions where specimens of *K. flavicollis* were collected, while there are 15 regions where specimens of *R. lucifugus* were collected. Concerning the localities of collection, for both species, the highest number of localities is in Southern Italy and in the two main Italian islands, Sicily and Sardinia. For *K. flavicollis,* the most represented regions are Sardinia (21 localities), Sicily (16 localities), Campania (12 localities) and Apulia (11 localities). For *R. lucifugus* the most represented regions are Basilicata (12 localities), Apulia (12 localities) and Campania (10 localities).

For *K. flavicollis,* there are six localities not mentioned in the study of Italian termites published in 2008 by Sbrenna and Micciarelli Sbrenna: Riomaggiore (Liguria), Fiscaglia (Emilia-Romagna), Carrara (Tuscany), Quartu Sant’Elena, Santu Lussurgiu and Sinnai (Sardinia). For *R. lucifugus* there are four localities not mentioned in the study of 2008: Camogli (Liguria), Barberino Tavernelle (Tuscany), Miranda (Molise) and Quartu Sant’Elena (Sardinia). In the study of 2008, no locality was reported for Molise; thus, the specimens from Miranda represent significant evidence on the diffusion of *R. lucifugus* in this Italian region.

A very interesting result is the presence in the collection of a new species, *K. italicus* sp. nov., with its typical dark pronotum, contained in jar 14 (locality Marina di Alberese, province of Grosseto), and probably also in jars I, II and IV. This species has been reported only in the Italian territory and is widespread in the regions of central Italy.

Concerning the specimens of foreign termites, the continent represented by the highest number of specimens is Africa.

The recovering and re-cataloguing of the valuable collection of Professor Antonio Springhetti represents not only the restoration and the rescue of a precious scientific and cultural asset but also a basis for future morphological, ultrastructural and molecular studies for phylogenetic, cladistic and geographical investigations.

Termites, a group of insects of high ecological relevance for recycling of organic matter, are also considered a serious biodeteriogenic agent and very detrimental for wooden artworks, old buildings and ancient books. In the case of the Springhetti Collection, termite specimens may actually become a valuable cultural and scientific asset, worthy of rescue, preservation and safeguard for future studies.

## Data Availability

Not applicable.

## Supplementary Materials

The following Supplementary Material is available online at https://www.mdpi.com/article/10.3390/insects12090793/s1, Tables S1–S31.
