# Supplementary material for: A Historical Collection of Termites in Ferrara: Recovery, Cataloguing and Geographical Analyses
Source: Insects. 2021 Sep 4;12(9):793. doi: 10.3390/insects12090793 (PMC8471403; doi:10.3390/insects12090793)
Supplement: Supplementary file 1 [file insects-12-00793-s001.zip › insects-1338536-supplementary.pdf]

*insects*

MDPI

*Supplementary Material*

# A Historical Collection of Termites in Ferrara: Recovery, Cataloguing and Geographical Analyses

**Davide Curci[1], Chiara Scapoli[1], Maria Gabriella Marchetti[1], Milvia Chicca[1], Marilena Leis[1], Chiara Beatrice Vicentini[1], Teresa Bonacci[2], Marco Pezzi[1*]**

[1] Department of Life Sciences and Biotechnology, University of Ferrara, Via L. Borsari 46, 44121, Ferrara, Italy; davide.curci@edu.unife.it (D.C.); chiara.scapoli@unife.it (C.S.); gabriella.marchetti@unife.it (M.G.M.) milvia.chicca@unife.it (M.C.); marilena.leis@unife.it (M.L.) chiara.vicentini@unife.it (C.B.V); marco.pezzi@unife.it (M.P.)

[2] Department of Biology, Ecology and Earth Sciences, University of Calabria, Via P. Bucci, 87036, Arcavacata di Rende, Cosenza, Italy; teresa.bonacci@unical.it (T.B.)

* Correspondence: marco.pezzi@unife.it

**Citation:** Curci, D.; Scapoli, C.; Marchetti, M.G.; Chicca, M.; Leis, M.; Vicentini, C.B.; Bonacci, T.; Pezzi, M. A Historical Collection of Termites in Ferrara: Recovery, Cataloguing and Geographical Analyses. *Insects* **2021**, *12*, 793. https://doi.org/10.3390/insects12090793

Academic Editor: Barbara Manachini and Stefano Vanin

Received: 28 July 2021
Accepted: 31 August 2021
Published: 4 September 2021

**Publisher's Note:** MDPI stays neutral with regard to jurisdictional claims in published maps and institutional affiliations.

**Copyright:** © 2021 by the authors. Submitted for possible open access publication under the terms and conditions of the Creative Commons Attribution (CC BY) license (https://creativecommons.org/licenses/by/4.0/).

**Table S1.** Termite specimens from Springhetti Collection. Jar 1: *Reticulitermes lucifugus*, laboratory specimens. Abbreviations: A, alates; E, ergatoids; N, neanids; P, pseudergates; S, soldiers; R, royals; W: workers. Number of individuals of each caste within brackets.

| Test tube code | Number of individuals | Castes |
|---|---|---|
| V1-P01 | 19 | E(9); W(10) |
| V1-P02 | 1 | R(1) |
| V1-P03 | 75 | S(1); W(74) |
| V1-P04 | 2 | A(2) |
| V1-P05 | 2 | S(2) |
| V1-P06 | 13 | S(6); W(7) |
| V1-P07 | 7 | S(7) |
| V1-P08 | 9 | W(9) |
| V1-P09 | 22 | N(22) |
| V1-P10 | 29 | N(29) |
| V1-P11 | 4 | N(4) |
| V1-P12 | 30 | N(30) |
| V1-P13 | 6 | S(6) |
| V1-P14 | 23 | E(5); N(7); W(11) |
| V1-P15 | 37 | N(37) |
| V1-P16 | 39 | S(21); W(18); |
| V1-P17 | 54 | W(54) |
| V1-P18 | 43 | E(9); N(26); W(8) |

**Table S2.** Termite specimens from Springhetti Collection. Jar 2: Italian termites, *Kalotermes flavicollis* (*K. flavicollis*), *Reticulitermes lucifugus* (*R. lucifugus*). *Specimens collected in the wild and afterwards kept in laboratory. All other abbreviations as in Table S1.

| Test tube code | Species | Number of individuals | Castes | Year of collection | Administrative Region | Municipality/Locality |
|---|---|---|---|---|---|---|
| V2-P01 | *K. flavicollis* | 30 | A(9); P(11); S(10); | -- | Emilia-Romagna | Ferrara |
| V2-P02 | *R. lucifugus* | 175 | N(1); S(6); W(168) | -- | Lazio | Antrodoco (Rieti) |
| V2-P03 | *R. lucifugus* | 133 | N(3); S(19); W(111) | 1987 | Emilia-Romagna | Bagnacavallo (Ravenna) |
| V2-P04 | *R. lucifugus* | 13 | S(10); W(3) | 1977 | Friuli-Venezia Giulia | Udine |
| V2-P05 | *K. flavicollis* | 5 | S(5) | 1976 | Liguria | Marinella Sarzana (Sarzana, La Spezia) |
| V2-P06 | *R. lucifugus* | 70 | A(70) | 1954 | Lazio | Roma |
| V2-P07 | *R. lucifugus* | 2 | A(2) | -- | Emilia-Romagna | Bagnacavallo (Ravenna) |
| V2-P08 | *R. lucifugus* | 8 | S(2); W(6) | 1988 | Liguria | Camogli (Genova) |
| V2-P09 | *R. lucifugus* | 72 | S(5); W(67) | 1966 | Tuscany | Livorno |
| V2-P10 | *K. flavicollis* | 41 | A(28); P(11); S(2) | -- | Emilia-Romagna | Ferrara |
| V2-P11 | *R. lucifugus* | 878 | S(9); W(869) | 1959 | Lombardy | Lodi |
| V2-P12 | *K. flavicollis* | 1 | A(1) | -- | Liguria | Manarola (Riomaggiore, La Spezia) |
| V2-P13 | *K. flavicollis* | 1 | A(1) | 1972 | Emilia-Romagna | Pineta di San Vitale (Ravenna) |
| V2-P14 | *K. flavicollis* | 18 | P(10); S(8) | -- | Emilia-Romagna | Ferrara |
| V2-P15 | *R. lucifugus* | 13 | S(6); W(7) | -- | Tuscany | Barberino di Mugello (Firenze) |
| V2-P16 | *R. lucifugus* | 22 | N(6); S(8); W(8) | 1966 | Tuscany | Pisa |
| V2-P17 | *R. lucifugus* | 15 | N(5); S(4); W(6) | -- | Lombardy | Lodi |
| V2-P18 | *R. lucifugus* | 16 | S(9); W(7) | -- | Tuscany | Barberino di Mugello (Firenze) |
| V2-P19 | *R. lucifugus* | 14 | S(1); W(13) | 1964 | Lazio | Istituto Patologia del Libro (Roma)* |
| V2-P20 | *R. lucifugus* | 14 | S(8); W(6) | 1958 | Marche | Castel Raimondo (Macerata) |
| V2-P21 | *R. lucifugus* | 102 | N(11); S(1); W(90) | 1964 | Emilia-Romagna | Salsomaggiore Terme (Parma) |
| V2-P22 | *K. flavicollis* | 29 | A(1); P(26); S(2) | 1975 | Emilia-Romagna | San Fortunato (Rimini) |
| V2-P23 | *K. flavicollis* | 11 | P(9); S(2) | 1974 | Emilia-Romagna | Masi Torello (Ferrara) |
| V2-P24 | *R. lucifugus* | 31 | S(5); W(26) | -- | Tuscany | Pineta del Tombolo (Grosseto) |
| V2-P25 | *R. lucifugus* | 4 | W(4) | 1977 | Tuscany | Lido di Camaiore (Camaiore, Lucca) |
| V2-P26 | *R. lucifugus* | 33 | S(4); W(29) | 1964 | Emilia-Romagna | Salsomaggiore Terme (Parma) |
| V2-P27 | *R. lucifugus* | 1 | W(1) | 1987 | Emilia-Romagna | Bagnacavallo (Ravenna) |
| V2-P28 | *R. lucifugus* | 23 | N(5); W(18) | -- | Tuscany | Barberino Val d'Elsa (Barberino Tavarnelle, Firenze) |
| V2-P29 | *R. lucifugus* | 22 | S(9); W(13) | 1971 | Tuscany | Bagni di Lucca (Lucca) |
| V2-P30 | *R. lucifugus* | 27 | A(2); N(2); S(5); W(18) | -- | Emilia-Romagna | Bagnacavallo (Ravenna)* |
| V2-P31 | *R. lucifugus* | 36 | S(13); W(23) | -- | Tuscany | Poggibonsi (Siena) |
| V2-P32 | *R. lucifugus* | 19 | S(9); W(10) | -- | Tuscany | San Donato (Reggello, Firenze) |
| V2-P33 | *R. lucifugus* | 46 | N(3); S(4); W(39) | 1987 | Emilia-Romagna | Bagnacavallo (Ravenna) |
| V2-P34 | *R. lucifugus* | 39 | A(2); S(7); W(30) | -- | Umbria | Todi (Perugia) |
| V2-P35 | *R. lucifugus* | 71 | S(13); W(58) | 1966 | Tuscany | Livorno |
| V2-P36 | *R. lucifugus* | 169 | W(169) | 1959 | Lombardy | Lodi |
| V2-P37 | *R. lucifugus* | 111 | S(7); W(104) | 1959 | Lombardy | Lodi |
| V2-P38 | *R. lucifugus* | 35 | S(9); W(26) | 1966 | Tuscany | Livorno |

**Table S3.** Termite specimens from Springhetti Collection. Jar 3: Italian termites, *R. lucifugus.* All abbreviations as in Tables S1 and S2.

| Test tube code | Number of individuals | Castes | Year of collection | Administrative Region | Municipality/Locality |
|---|---|---|---|---|---|
| V3-P01 | 424 | N(108); S(1); W(315) | 1953 | Apulia | Gallipoli (Lecce) |
| V3-P02 | 18 | S(1); W(17) | 1973 | Apulia | Manfredonia (Foggia) |
| V3-P03 | 47 | S(10); W(37) | 1975 | Apulia | Leuca (Lecce) |
| V3-P04 | 22 | N(9); S(13) | 1983 | Apulia | Squinzano (Lecce) |
| V3-P05 | 14 | S(1); W(13) | 1965 | Apulia | Rocchetta Sant'Antonio (Foggia) |
| V3-P06 | 33 | S(1); W(32) | 1973 | Apulia | Bosco Quarto (Monte Sant'Angelo, Foggia) |
| V3-P07 | 31 | W(31) | 1973 | Apulia | Monte Sant'Angelo (Foggia) |
| V3-P08 | 19 | E(2); S(1); W(16) | -- | Apulia | Squinzano (Lecce)* |
| V3-P09 | 38 | W(38) | 1973 | Apulia | Monte Sant'Angelo (Foggia) |
| V3-P10 | 4 | S(1); W(3) | 1973 | Apulia | Peschici (Foggia) |
| V3-P11 | 15 | S(2); W(13) | 1973 | Apulia | Monte Sant'Angelo (Foggia) |
| V3-P12 | 47 | S(2); W(45) | 1973 | Apulia | Bosco Quarto (Monte Sant'Angelo, Foggia) |
| V3-P13 | 99 | S(1); W(98) | 1973 | Apulia | Funno (San Giovanni Rotondo, Foggia) |
| V3-P14 | 11 | S(2); W(9) | 1973 | Apulia | Funno (San Giovanni Rotondo, Foggia) |
| V3-P15 | 43 | N(27); S(1); W(15) | 1973 | Apulia | Monte Sant'Angelo (Foggia) |
| V3-P16 | 77 | N(1); W(76) | 1973 | Apulia | Monte Sant'Angelo (Foggia) |
| V3-P17 | 13 | S(1); W(12) | 1964 | Apulia | Foggia |
| V3-P18 | 64 | N(36); S(5); W(23) | 1973 | Apulia | Mattinata (Foggia) |
| V3-P19 | 55 | S(2); W(53) | 1973 | Apulia | Mattinata (Foggia) |
| V3-P20 | 65 | S(4); W(61) | 1975 | Apulia | Galatone (Lecce) |
| V3-P21 | 121 | N(15); S(10); W(96) | 1973 | Apulia | Monte Sant'Angelo (Foggia) |
| V3-P22 | 40 | S(3); W(37) | 1973 | Apulia | San Giovanni Rotondo (Foggia) |
| V3-P23 | 24 | S(3); W(21) | 1960 | Basilicata | Maratea (Potenza) |
| V3-P24 | 11 | S(11) | 1964 | Basilicata | Melfi (Potenza) |
| V3-P25 | 12 | N(5); W(7) | 1964 | Basilicata | Garaguso (Matera) |
| V3-P26 | 1 | W(1) | -- | Calabria | Rende (Cosenza) |
| V3-P27 | 9 | N(8); W(1) | -- | Calabria | Belvedere Spinello (Crotone) |
| V3-P28 | 3 | W(3) | -- | Calabria | Pellegrina (Bagnara di Calabria, Reggio Calabria) |
| V3-P29 | 3 | S(1); W(2) | -- | Basilicata | Rapone (Potenza) |
| V3-P30 | 40 | S(12); W(28) | 1964 | Basilicata | Lagonegro (Potenza) |
| V3-P31 | 4 | S(1); W(3) | 1964 | Basilicata | Metaponto (Bernalda, Matera) |
| V3-P32 | 6 | W(6) | 1964 | Basilicata | Lauria (Potenza) |
| V3-P33 | 4 | S(2); W(2) | -- | Calabria | Belvedere Spinello (Crotone) |
| V3-P34 | 13 | N(2); S(1); W(10) | 1964 | Basilicata | Acerenza (Potenza) |
| V3-P35 | 14 | S(7); W(7) | 1964 | Basilicata | Trecchina (Potenza) |
| V3-P36 | 3 | S(1); W(2) | 1980 | Calabria | Praia a mare (Cosenza) |
| V3-P37 | 22 | S(9); W(13) | 1964 | Basilicata | Rionero in Vulture (Potenza) |
| V3-P38 | 18 | S(5); W(13) | 1964 | Basilicata | Moliterno (Potenza) |
| V3-P39 | 18 | S(16); W(2) | 1964 | Basilicata | Melfi (Potenza) |
| V3-P40 | 26 | S(9); W(17) | 1964 | Basilicata | Melfi (Potenza) |
| V3-P41 | 53 | S(2); W(51) | 1964 | Basilicata | Latronico (Potenza) |
| V3-P42 | 29 | S(5); W(24) | 1964 | Basilicata | Melfi (Potenza) |
| V3-P43 | 93 | S(6); W(87) | 1975 | Apulia | Squinzano (Lecce) |
| V3-P44 | 162 | S(1); W(161) | 1968 | Apulia | Manduria (Taranto) |

**Table S4.** Termite specimens from Springhetti Collection. Jar 4: Italian termites, *R. lucifugus.* All abbreviations as in Table S1.

| Test tube code | Number of individuals | Castes | Year of collection | Administrative Region | Municipality/Locality |
|---|---|---|---|---|---|
| V4-P01 | 11 | A(9); W(2) | 1981 | Campania | Ischia (Napoli) |
| V4-P02 | 7 | W(7) | 1981 | Campania | Barano d'Ischia (Napoli) |
| V4-P03 | 8 | S(1); W(7) | 1961 | Campania | Procida (Napoli) |
| V4-P04 | 15 | S(2); W(13) | -- | Campania | Serrara Fontana (Napoli) |
| V4-P05 | 29 | N(8); S(7); W(14) | -- | Campania | Ischia (Napoli) |
| V4-P06 | 50 | S(9); W(41) | 1964 | Campania | Pertosa (Salerno) |
| V4-P07 | 18 | W(18) | -- | Molise | Miranda (Isernia) |
| V4-P08 | 14 | N(9); S(5) | -- | Campania | Ischia (Napoli) |
| V4-P09 | 13 | N(6); W(7) | 1961 | Campania | Serrara Fontana (Napoli) |
| V4-P10 | 53 | N(51); W(2) | -- | Campania | Ischia (Napoli) |
| V4-P11 | 38 | S(6); W(32) | 1964 | Campania | Maddaloni (Caserta) |
| V4-P12 | 243 | S(6); W(237) | 1965 | Campania | Lapio (Avellino) |
| V4-P13 | 54 | N(11); S(6);W(37) | 1964 | Campania | Ariano Irpino (Avellino) |
| V4-P14 | 4 | A(4) | -- | Campania | Monteforte Irpino (Avellino) |
| V4-P15 | 22 | S(2); W(20) | 1981 | Campania | Testaccio d'Ischia (Barano d'Ischia, Napoli) |
| V4-P16 | 15 | S(6); W(9) | 1964 | Campania | Fisciano (Salerno) |
| V4-P17 | 18 | N(4); S(6); W(8) | 1982 | Campania | Ischia (Napoli) |

**Table S5.** Termite specimens from Springhetti Collection. Jar 5: Italian termites, *R. lucifugus*. All abbreviations as in Tables S1 and S2.

| Test tube code | Number of individuals | Castes | Year of collection | Administrative Region | Municipality/Locality |
|---|---|---|---|---|---|
| V5-P01 | 92 | S(11); W(81) | 1980 | Sardinia | Cala Gonone (Dorgali, Nuoro) |
| V5-P02 | 57 | S(3); W(54) | -- | Sardinia | Morgongiori (Oristano) |
| V5-P03 | 98 | S(7); W(91) | -- | Sardinia | Spiaggia di Is Mortorius (Quartu Sant'Elena, Cagliari) |
| V5-P04 | 45 | N(1); W(44) | 1965 | Sardinia | Campu Omu (Sinnai, Cagliari) |
| V5-P05 | 141 | S(9); W(132) | -- | Sardinia | Carbonia (Sud Sardegna) |
| V5-P06 | 23 | W(23) | -- | Sardinia | Sennori (Sassari) |
| V5-P07 | 5 | A(5) | -- | Sardinia | Pula (Cagliari)* |
| V5-P08 | 82 | W(82) | 1964 | Sardinia | Pula (Cagliari) |
| V5-P09 | 44 | S(2); W(42) | 1980 | Sardinia | Sennori (Sassari) |
| V5-P10 | 129 | S(26); W(103) | 1964 | Sardinia | -- |
| V5-P11 | 55 | S(9); W(46) | -- | Sardinia | Oliena (Nuoro) |
| V5-P12 | 3 | S(3) | 1963 | Sicily | Caltagirone (Catania) |
| V5-P13 | 2 | S(2) | -- | Sicily | Polizzi Generosa (Palermo)* |
| V5-P14 | 1 | W(1) | 1963 | Sicily | Pantano (Ribera, Agrigento) |
| V5-P15 | 23 | S(1); W(22) | 1963 | Sicily | Monserrato (Licata, Agrigento) |
| V5-P16 | 70 | N(10); W(48); S(12) | 1965 | Sicily | Pantelleria (Trapani) |
| V5-P17 | 15 | W(15) | 1963 | Sicily | Polizzi Generosa (Palermo) |
| V5-P18 | 8 | S(8) | -- | Sicily | Bagheria (Palermo) |
| V5-P19 | 10 | S(5); W(5) | 1963 | Sicily | Salina (Messina) |
| V5-P20 | 15 | W(15) | 1963 | Sicily | Porto Empedocle (Agrigento) |
| V5-P21 | 22 | N(1); S(5); W(16) | 1975 | Sicily | Polizzi Generosa (Palermo) |
| V5-P22 | 67 | N(1); S(10); W(56) | 1965 | Sicily | Pantelleria (Trapani) |
| V5-P23 | 101 | N(26); W(58); S(17) | 1965 | Sicily | Pantelleria (Trapani) |
| V5-P24 | 99 | N(1); S(26); W(72) | 1965 | Sicily | Pantelleria (Trapani) |
| V5-P25 | 148 | N(46); S(14); W(88) | 1965 | Sicily | Pantelleria (Trapani) |

**Table S6.** Termite specimens from Springhetti Collection. Jar 6: Italian termites, *K. flavicollis* from Sardinia. SE, secondary reproductives. All abbreviations as in Table S1.

| Test tube code | Number of individuals | Castes | Year of collection | Municipality/Locality |
|---|---|---|---|---|
| V6-P01 | 11 | S(11) | 1974 | Sennori (Sassari) |
| V6-P02 | 17 | P(7); S(10) | 1975 | Sennori (Sassari) |
| V6-P03 | 23 | S(23) | 1994 | San Giovanni Suergiu (Sud Sardegna) |
| V6-P04 | 7 | S(7) | 1973 | Sennori (Sassari) |
| V6-P05 | 20 | A(1); N(1); P(15); S(3) | 1965 | Sant'Antioco (Sud Sardegna) |
| V6-P06 | 10 | A(10) | -- | Sant'Antioco (Sud Sardegna) |
| V6-P07 | 17 | P(7); S(10) | -- | San Giovanni Suergiu (Sud Sardegna) |
| V6-P08 | 20 | P(10); S(10) | -- | Sant'Antioco (Sud Sardegna) |
| V6-P09 | 11 | S(11) | 1975 | Sennori (Sassari) |
| V6-P10 | 8 | S(8) | -- | Sant'Antioco (Sud Sardegna) |
| V6-P11 | 10 | S(10) | 1975 | Sennori (Sassari) |
| V6-P12 | 10 | A(10) | -- | San Giovanni Suergiu (Sud Sardegna) |
| V6-P13 | 10 | P(8); S(2) | -- | Villasimius (Sud Sardegna) |
| V6-P14 | 22 | P(3); S(19) | -- | Pula (Cagliari) |
| V6-P15 | 132 | A(132) | -- | San Giovanni Suergiu (Sud Sardegna) |
| V6-P16 | 110 | S(110) | 1994 | San Giovanni Suergiu (Sud Sardegna) |
| V6-P17 | 34 | N(4); P(9); S(19); R(1) | 1966 | San Giovanni Suergiu (Sud Sardegna) |
| V6-P18 | 32 | A(13); N(1); P(6); S(12); | -- | San Giovanni Suergiu (Sud Sardegna) |
| V6-P19 | 15 | P(6); S(9) | -- | San Giovanni Suergiu (Sud Sardegna) |
| V6-P20 | 10 | P(10) | -- | Santu Lussurgiu (Oristano) |
| V6-P21 | 18 | P(6); S(12) | -- | San Giovanni Suergiu (Sud Sardegna) |
| V6-P22 | 10 | A(10) | -- | San Giovanni Suergiu (Sud Sardegna) |
| V6-P23 | 24 | P(8); S(15);SE(1) | 1966 | San Giovanni Suergiu (Sud Sardegna) |
| V6-P24 | 31 | A(6); P(12); S(13) | -- | San Giovanni Suergiu (Sud Sardegna) |
| V6-P25 | 29 | A(5); P(10); S(14) | -- | San Giovanni Suergiu (Sud Sardegna) |
| V6-P26 | 27 | A(1); P(15); S(11) | -- | San Giovanni Suergiu (Sud Sardegna) |
| V6-P27 | 9 | A(1); P(6); S(2) | -- | San Giovanni Suergiu (Sud Sardegna) |
| V6-P28 | 10 | A(10) | -- | San Giovanni Suergiu (Sud Sardegna) |
| V6-P29 | 12 | P(4); S(8) | -- | San Giovanni Suergiu (Sud Sardegna) |
| V6-P30 | 29 | N(1); P(9); S(19) | -- | San Giovanni Suergiu (Sud Sardegna) |
| V6-P31 | 47 | P(23); S(24) | -- | San Giovanni Suergiu (Sud Sardegna) |
| V6-P32 | 84 | P(13); S(71) | 1966 | San Giovanni Suergiu (Sud Sardegna) |
| V6-P33 | 24 | P(14); S(10) | -- | San Giovanni Suergiu (Sud Sardegna) |
| V6-P34 | 25 | P(14); S(11) | -- | San Giovanni Suergiu (Sud Sardegna) |
| V6-P35 | 28 | P(11); S(17) | -- | San Giovanni Suergiu (Sud Sardegna) |
| V6-P36 | 45 | P(26); S(19) | -- | San Giovanni Suergiu (Sud Sardegna) |
| V6-P37 | 51 | P(28); S(23) | 1966 | San Giovanni Suergiu (Sud Sardegna) |
| V6-P38 | 12 | P(1); S(11) | -- | San Giovanni Suergiu (Sud Sardegna) |
| V6-P39 | 28 | A(3); N(2); P(21); S(2); | -- | San Giovanni Suergiu (Sud Sardegna) |
| V6-P40 | 20 | P(10); S(10) | -- | San Giovanni Suergiu (Sud Sardegna) |
| V6-P41 | 44 | N(6); P(15); S(23) | 1966 | San Giovanni Suergiu (Sud Sardegna) |
| V6-P42 | 65 | S(65) | -- | San Giovanni Suergiu (Sud Sardegna) |
| V6-P43 | 44 | A(44) | -- | San Giovanni Suergiu (Sud Sardegna) |
| V6-P44 | 55 | A(2); P(30); S(23) | -- | San Giovanni Suergiu (Sud Sardegna) |
| V6-P45 | 58 | A(1); P(37); S(20) | -- | San Giovanni Suergiu (Sud Sardegna) |
| V6-P46 | 23 | A(13); N(1); P(5); S(4) | -- | San Giovanni Suergiu (Sud Sardegna) |

**Table S7.** Termite specimens from Springhetti Collection. Jar 7: Italian termites, *K. flavicollis* from Sardinia. All abbreviations as in Tables S1 and S2.

| Test tube code | Number of individuals | Castes | Year of collection | Municipality/Locality |
|---|---|---|---|---|
| V7-P01 | 40 | P(25); S(15) | -- | Campu Omu (Sinnai, Cagliari) |
| V7-P02 | 20 | R(1); S(19) | -- | Cagliari |
| V7-P03 | 3 | P(3) | -- | Domusnovas (Sud Sardegna) |
| V7-P04 | 37 | A(5); P(17); S(15) | 1965 | Cagliari |
| V7-P05 | 12 | P(4); S(8) | 1975 | Sennori (Sassari) |
| V7-P06 | 30 | P(15); S(15) | -- | Campu Omu (Sinnai, Cagliari) |
| V7-P07 | 11 | S(11) | -- | Sennori (Sassari) |
| V7-P08 | 22 | S(22) | -- | Cagliari |
| V7-P09 | 64 | P(59); S(5) | -- | Domusnovas (Sud Sardegna) |
| V7-P10 | 45 | A(12); P(21); S(12) | 1965 | Las Plassas (Sud Sardegna) |
| V7-P11 | 37 | A(5); N(2); P(7); S(23) | -- | Cagliari |
| V7-P12 | 19 | P(11); S(8) | -- | Campu Omu (Sinnai, Cagliari) |
| V7-P13 | 17 | A(17) | 1966 | Barumini (Sud Sardegna) |
| V7-P14 | 40 | P(40) | 1966 | Oristano |
| V7-P15 | 10 | S(10) | 1975 | Sennori (Sassari) |
| V7-P16 | 7 | S(7) | -- | Sennori (Sassari) |
| V7-P17 | 37 | P(34); S(3) | -- | Portoscuso (Sud Sardegna) |
| V7-P18 | 11 | A(5); P(4); S(2) | 1965 | Barumini (Sud Sardegna) |
| V7-P19 | 5 | A(5) | -- | Domusnovas (Sud Sardegna) |
| V7-P20 | 27 | P(13); S(14) | -- | Las Plassas (Sud Sardegna) |
| V7-P21 | 15 | P(15) | -- | Carbonia (Sud Sardegna) |
| V7-P22 | 5 | P(1); S(4) | -- | Pula (Cagliari)* |
| V7-P23 | 123 | P(101); R(2); S(20) | 1965 | Teulada (Sud Sardegna) |
| V7-P24 | 19 | P(14); R(4); S(1) | -- | Teulada (Sud Sardegna) |
| V7-P25 | 73 | P(60); S(13) | -- | Portoscuso (Sud Sardegna) |
| V7-P26 | 61 | A(30); P(25); S(6) | -- | Oristano |
| V7-P27 | 38 | N(1); P(13); S(24) | -- | Burcei (Sud Sardegna) |
| V7-P28 | 24 | P(13); R(2); S(9) | -- | Campu Omu (Sinnai, Cagliari) |
| V7-P29 | 79 | A(20); N(2); P(57) | 1966 | Teulada (Sud Sardegna) |
| V7-P30 | 27 | P(25); S(2) | -- | Las Plassas (Sud Sardegna) |
| V7-P31 | 8 | P(5); S(3) | 1974 | Carloforte (Sud Sardegna) |
| V7-P32 | 25 | P(24); S(1) | -- | Domusnovas (Sud Sardegna) |
| V7-P33 | 34 | P(22); S(12) | -- | Bonarcado (Oristano) |
| V7-P34 | 19 | A(6); P(11); S(2) | -- | Oristano |
| V7-P35 | 11 | S(11) | 1974 | Sennori (Sassari) |
| V7-P36 | 73 | N(4); P(69) | -- | Ulassai (Nuoro) |
| V7-P37 | 78 | A(33); P(36); S(9) | 1966 | Oristano |
| V7-P38 | 24 | P(6); S(18) | -- | Bonarcado (Oristano)* |
| V7-P39 | 6 | A(6) | -- | Bonarcado (Oristano) |
| V7-P40 | 20 | S(20) | 1973 | Sennori (Sassari) |
| V7-P41 | 33 | S(33) | 1973 | Sennori (Sassari) |
| V7-P42 | 73 | P(61); S(12) | -- | Spiaggia di Is Mortorius (Quartu Sant'Elena, Cagliari) |
| V7-P43 | 11 | A(6); P(2); S(3) | 1966 | Gonnesa (Sud Sardegna) |
| V7-P44 | 14 | A(8); P(2); S(4) | 1965 | Villasimius (Sud Sardegna) |
| V7-P45 | 26 | P(12); S(14) | -- | Campu Omu (Sinnai, Cagliari) |
| V7-P46 | 9 | S(9) | -- | Sennori (Sassari) |
| V7-P47 | 37 | P(27); S(10) | 1964 | Bonarcado (Oristano) |
| V7-P48 | >100 | Eggs | -- | Pula (Cagliari) |

| | | | | |
|---|---|---|---|---|
| V7-P49 | 6 | P(4); R(2) | -- | Burcei (Sud Sardegna) |

**Table S8.** Termite specimens from Springhetti Collection. Jar 8: Italian termites, *K. flavicollis* from Sardinia. All abbreviations as in Tables S1 and S2.

| Test tube code | Number of individuals | Castes | Year of collection | Municipality/Locality |
|---|---|---|---|---|
| V8-P01 | 8 | P(3); S(5) | -- | Sennori (Sassari) |
| V8-P02 | 11 | P(6); S(5) | -- | Sennori (Sassari) |
| V8-P03 | 18 | P(7); S(11) | -- | Sennori (Sassari) |
| V8-P04 | 11 | P(4); S(7) | -- | Sennori (Sassari) |
| V8-P05 | 10 | S(10) | 1975 | Sennori (Sassari) |
| V8-P06 | 10 | A(10) | -- | Sennori (Sassari)* |
| V8-P07 | 9 | S(9) | 1975 | Sennori (Sassari) |
| V8-P08 | 2 | S(2) | -- | Sennori (Sassari) |
| V8-P09 | 14 | S(14) | 1976 | Sennori (Sassari) |
| V8-P10 | 15 | P(9); S(6) | -- | Sennori (Sassari) |
| V8-P11 | 21 | P(11); S(10) | -- | Sennori (Sassari) |
| V8-P12 | 15 | P(6); S(9) | -- | Sennori (Sassari) |
| V8-P13 | 25 | P(17); S(8) | -- | Sennori (Sassari) |
| V8-P14 | 38 | P(20); S(18) | 1966 | San Giovanni Suergiu (Sud Sardegna) |
| V8-P15 | 11 | S(11) | -- | San Giovanni Suergiu (Sud Sardegna) |
| V8-P16 | 15 | S(15) | -- | Sennori (Sassari) |
| V8-P17 | 14 | S(14) | -- | Sant'Antioco (Sud Sardegna) |
| V8-P18 | 57 | A(1); P(29); S(27) | -- | San Giovanni Suergiu (Sud Sardegna) |
| V8-P19 | 14 | P(4); S(10) | -- | San Giovanni Suergiu (Sud Sardegna) |
| V8-P20 | 48 | A(2); P(18); S(28) | 1966 | Cagliari* |

**Table S9.** Termite specimens from Springhetti Collection. Jar 9: Italian termites, *K. flavicollis* from Sicily. All abbreviations as in Table S1.

| Test tube code | Number of individuals | Castes | Year of collection | Municipality/Locality |
|---|---|---|---|---|
| V9-P01 | 14 | A(7); P(6); S(1) | 1975 | Polizzi Generosa (Palermo) |
| V9-P02 | 6 | S(6) | -- | Palermo |
| V9-P03 | 2 | P(2) | -- | Bagheria (Palermo) |
| V9-P04 | 1 | R(1) | -- | Favara (Agrigento) |
| V9-P05 | 3 | P(3) | 1963 | Ribera (Agrigento) |
| V9-P06 | 5 | A(5) | -- | Palermo? |
| V9-P07 | 2 | A(1); P(1) | -- | Rosolini (Siracusa) |
| V9-P08 | 15 | P(6); S(9) | -- | Polizzi Generosa (Palermo) |
| V9-P09 | 7 | P(7) | -- | Siracusa |
| V9-P10 | 55 | A(7); N(3); P(26); S(19) | -- | Polizzi Generosa (Palermo) |
| V9-P11 | 14 | A(2); P(12) | 1963 | Agrigento |
| V9-P12 | 37 | A(32); P(4); S(1) | 1967 | Messina |
| V9-P13 | 2 | A(2) | 1978 | Castelvetrano (Trapani) |
| V9-P14 | 14 | P(7); S(7) | -- | Polizzi Generosa (Palermo) |
| V9-P15 | 33 | P(22); S(11) | 1975 | Polizzi Generosa (Palermo) |
| V9-P16 | 25 | P(15); S(10) | -- | Palermo |
| V9-P17 | 5 | P(4); S(1) | 1963 | Palma di Montechiaro (Agrigento) |
| V9-P18 | 6 | A(6) | 1975 | Polizzi Generosa (Palermo) |
| V9-P19 | 17 | P(11); S(6) | -- | Polizzi Generosa (Palermo) |
| V9-P20 | 14 | P(9); S(5) | -- | Polizzi Generosa (Palermo) |
| V9-P21 | 18 | P(12); S(6) | -- | Polizzi Generosa (Palermo) |
| V9-P22 | 23 | A(11); P(6); S(60) | -- | Polizzi Generosa (Palermo) |
| V9-P23 | 32 | P(18); S(14) | 1975 | Polizzi Generosa (Palermo) |
| V9-P24 | 19 | P(8); S(11) | -- | Polizzi Generosa (Palermo) |
| V9-P25 | 3 | P(2); S(1) | 1975 | Polizzi Generosa (Palermo) |
| V9-P26 | 25 | P(17); S(8) | -- | Polizzi Generosa (Palermo) |
| V9-P27 | 1 | R(1) | 1963 | Siculiana (Agrigento) |
| V9-P28 | 14 | A(6); P(3); S(5) | -- | Polizzi Generosa (Palermo) |
| V9-P29 | 19 | A(6); N(2); P(4); S(7) | 1976 | Polizzi Generosa (Palermo) |
| V9-P30 | 58 | A(1); P(48); S(9) | 1975 | Polizzi Generosa (Palermo) |
| V9-P31 | 34 | P(12); S(22) | -- | Palermo |
| V9-P32 | 33 | P(11); S(22) | 1965 | Palermo |
| V9-P33 | 30 | A(9); P(10); S(11) | -- | Polizzi Generosa (Palermo) |
| V9-P34 | 56 | A(1); P(45); S(10) | 1975 | Polizzi Generosa (Palermo) |
| V9-P35 | 21 | P(17); S(4) | 1963 | Caduta (Licata, Agrigento) |
| V9-P36 | 2 | A(1); P(1) | 1963 | Monserra (Licata, Agrigento) |

**Table S10.** Termite specimens from Springhetti Collection. Jar 10: Italian termites, *K. flavicollis* from minor islands of Sicily. All abbreviations as in Table S1.

| Test tube code | Number of individuals | Castes | Year of collect ion | Municipality/Locality |
|---|---|---|---|---|
| V10-P01 | 76 | P(31); S(45) | 1965 | Linosa (Agrigento) |
| V10-P02 | 331 | P(172); S(159) | -- | Linosa (Agrigento) |
| V10-P03 | 221 | A(218); P(3) | -- | Linosa (Agrigento) |
| V10-P04 | 19 | A(19) | -- | Linosa (Agrigento) |
| V10-P05 | 32 | P(13); S(19) | 1965 | Linosa (Agrigento) |
| V10-P06 | 34 | A(19); P(8); S(7) | 1965 | Linosa (Agrigento) |
| V10-P07 | 57 | P(25); S(32) | 1965 | Linosa (Agrigento) |
| V10-P08 | 34 | P(12); S(22) | 1965 | Linosa (Agrigento) |
| V10-P09 | 48 | P(13); S(35) | 1965 | Linosa (Agrigento) |
| V10-P10 | 16 | P(9); R(2); S(5) | 1965 | Linosa (Agrigento) |
| V10-P11 | 51 | A(3); P(36); S(12) | 1965 | Linosa (Agrigento) |
| V10-P12 | 14 | A(1); P(8); S(5) | -- | Linosa (Agrigento) |
| V10-P13 | 18 | P(10); S(8) | 1965 | Linosa (Agrigento) |
| V10-P14 | 69 | P(51); S(18) | -- | Linosa (Agrigento) |
| V10-P15 | 18 | A(18) | 1965 | Linosa (Agrigento) |
| V10-P16 | 24 | P(20); S(4) | 1965 | Linosa (Agrigento) |
| V10-P17 | 9 | A(9) | -- | Linosa (Agrigento) |
| V10-P18 | 67 | P(25); S(42) | 1965 | Linosa (Agrigento) |
| V10-P19 | 46 | A(33); S(13) | 1965 | Lampedusa (Agrigento) |
| V10-P20 | 22 | A(1); P(11); S(10) | -- | Lampedusa (Agrigento) |
| V10-P21 | 9 | A(3); P(4); S(2) | -- | Lampedusa (Agrigento) |
| V10-P22 | 12 | A(1); P(1); S(10) | -- | Lampedusa (Agrigento) |
| V10-P23 | 23 | S(23) | 1965 | Lampedusa (Agrigento) |
| V10-P24 | 24 | P(17); S(7) | -- | Lampedusa (Agrigento) |
| V10-P25 | 22 | P(12); S(10) | -- | Lampedusa (Agrigento) |
| V10-P26 | 18 | A(7); P(4); S(7) | 1965 | Lampedusa (Agrigento) |
| V10-P27 | 18 | A(4); N(2); P(8); S(4) | 1965 | Pantelleria (Trapani) |
| V10-P28 | 19 | P(10); S(9) | -- | Pantelleria (Trapani) |

**Table S11.** Termite specimens from Springhetti Collection. Jar 11: Italian termites, *K. flavicollis* from Apulia. All abbreviations as in Table S1.

| Test tube code | Number of individuals | Castes | Year of collection | Municipality/Locality |
|---|---|---|---|---|
| V11-P01 | 3 | P(2); S(1) | 1973 | Mattinata (Foggia) |
| V11-P02 | 1 | P(1) | 1973 | Manfredonia (Foggia) |
| V11-P03 | 9 | P(8); A(1) | 1973 | Mattinata (Foggia) |
| V11-P04 | 13 | A(1); P(10); S(2) | 1973 | Vico del Gargano (Foggia) |
| V11-P05 | 7 | P(7) | 1975 | Galatone (Lecce) |
| V11-P06 | 22 | A(7); P(7); S(8) | -- | Squinzano (Lecce) |
| V11-P07 | 10 | P(10) | 1971 | Squinzano (Lecce) |
| V11-P08 | 1 | A(1) | 1975 | Leuca (Lecce) |
| V11-P09 | 3 | P(2); S(1) | 1973 | Posta Padovano (San Giovanni Rotondo, Foggia) |
| V11-P10 | 11 | P(10); S(1) | 1973 | Mattinata (Foggia) |
| V11-P11 | 13 | S(13) | 1971 | Squinzano (Lecce) |
| V11-P12 | 15 | A(8); S(7) | -- | Squinzano (Lecce) |
| V11-P13 | 30 | P(25); S(5) | 1975 | Andrano (Lecce) |
| V11-P14 | 12 | P(6); S(6) | -- | Squinzano (Lecce) |
| V11-P15 | 9 | P(9) | 1973 | Manfredonia (Foggia) |
| V11-P16 | 9 | S(9) | 1954 | Gallipoli (Lecce) |
| V11-P17 | 2 | A(1); P(1) | 1973 | Manfredonia (Foggia) |
| V11-P18 | 26 | P(26) | 1973 | Manfredonia (Foggia) |
| V11-P19 | 5 | P(5) | 1973 | Manfredonia (Foggia) |
| V11-P20 | 8 | P(7); S(1) | 1973 | Posta Padovano (San Giovanni Rotondo, Foggia) |
| V11-P21 | 6 | P(5); S(1) | 1973 | Manfredonia (Foggia) |
| V11-P22 | 10 | P(8); S(2) | 1973 | Manfredonia (Foggia) |
| V11-P23 | 3 | P(3) | 1973 | Monte Sant'Angelo (Foggia) |
| V11-P24 | 18 | A(18) | 1976 | Squinzano (Lecce) |
| V11-P25 | 7 | S(7) | -- | Squinzano (Lecce) |
| V11-P26 | 1 | S(1) | 1968 | Manduria (Taranto) |
| V11-P27 | 10 | P(9); S(1) | 1973 | Mattinata (Foggia) |
| V11-P28 | 4 | P(3); S(1) | 1973 | Manfredonia (Foggia) |
| V11-P29 | 5 | P(5) | 1968 | Manduria (Taranto) |
| V11-P30 | 11 | P(10); S(1) | 1973 | San Giovanni Rotondo (Foggia) |
| V11-P31 | 3 | P(3) | 1973 | Manfredonia (Foggia) |
| V11-P32 | 21 | P(20); S(1) | 1973 | Manfredonia (Foggia) |
| V11-P33 | 2 | A(1); P(1) | 1973 | Manfredonia (Foggia) |
| V11-P34 | 5 | P(5) | 1973 | San Giovanni Rotondo (Foggia) |
| V11-P35 | 3 | P(3) | 1973 | Mattinata (Foggia) |
| V11-P36 | 6 | P(6) | 1973 | Manfredonia (Foggia) |
| V11-P37 | 11 | P(10); S(1) | 1973 | Manfredonia (Foggia) |
| V11-P38 | 29 | A(6); P(6); S(17) | 1975 | Squinzano (Lecce) |
| V11-P39 | 9 | P(9) | 1973 | Mattinata (Foggia) |
| V11-P40 | 13 | P(13) | 1973 | Mattinata (Foggia) |
| V11-P41 | 18 | P(16); S(2) | 1973 | Mattinata (Foggia) |
| V11-P42 | 28 | A(10); P(8); S(10) | -- | Squinzano (Lecce) |
| V11-P43 | 38 | P(37); S(1) | 1973 | Mattinata (Foggia) |
| V11-P44 | 6 | P(6) | 1973 | San Giovanni Rotondo (Foggia) |
| V11-P45 | 2 | P(2) | 1973 | Manfredonia (Foggia) |
| V11-P46 | 1 | S(1) | 1973 | Manfredonia (Foggia) |
| V11-P47 | 24 | A(23); P(1) | 1953 | Gallipoli (Lecce) |

| | | | | |
|---|---|---|---|---|
| V11-P48 | 36 | P(34); S(2) | 1973 | Mattinata (Foggia) |
| V11-P49 | 27 | P(26); S(1) | 1973 | Mattinata (Foggia) |
| V11-P50 | 319 | A(9); N(1); P(304); S(5) | 1953 | Gallipoli (Lecce) |
| V11-P51 | 96 | P(90); S(6) | 1975 | Squinzano (Lecce) |

**Table S12.** Termite specimens from Springhetti Collection. Jar 12: Italian termites, *K. flavicollis*. All abbreviations as in Table S1.

| Test tube code | Number of individuals | Castes | Year of collection | Administrative Region | Municipality/Locality |
|---|---|---|---|---|---|
| V12-P01 | 5 | P(5) | -- | Calabria | Belvedere di Spinello (Crotone) |
| V12-P02 | 33 | P(20); S(13) | 1976 | Calabria | Spilinga (Vibo Valentia) |
| V12-P03 | 108 | A(56); N(14); P(32); S(6) | 1974 | Calabria | Paola (Cosenza) |
| V12-P04 | 2 | A(1); P(1) | -- | Calabria | Pellegrina (Bagnara Calabra, Reggio Calabria) |
| V12-P05 | 5 | P(1); S(4) | -- | Calabria | Belvedere di Spinello (Crotone) |
| V12-P06 | 42 | A(1); P(28); S(13) | 1964 | Basilicata | Maratea (Potenza) |
| V12-P07 | 7 | P(6); S(1) | 1980 | Basilicata | Oliveto Lucano (Matera) |
| V12-P08 | 9 | A(2); P(4); S(3) | 1964 | Basilicata | Montalbano Jonico (Matera) |
| V12-P09 | 11 | P(11) | 1964 | Basilicata | Bernalda (Matera) |
| V12-P10 | 5 | S(5) | -- | Basilicata | Montalbano Jonico (Matera) |

**Table S13.** Termite specimens from Springhetti Collection. Jar 13: Italian termites, *K. flavicollis* from Campania. All abbreviations as in Tables S1 and S2.

| Test tube code | Number of individuals | Castes | Year of collection | Municipality/Locality |
|---|---|---|---|---|
| V13-P01 | 7 | P(6); S(1) | 1964 | Telese Terme (Benevento) |
| V13-P02 | 6 | P(3); S(3) | 1981 | Ischia (Napoli) |
| V13-P03 | 8 | A(2); P(6) | 1964 | Fisciano (Salerno) |
| V13-P04 | 1 | R(1) | 1980 | Barano d'ischia (Napoli) |
| V13-P05 | 11 | A(11) | 1964 | Capua (Caserta) |
| V13-P06 | 1 | R(1) | 1981 | Ischia (Napoli) |
| V13-P07 | 6 | P(3); S(3) | 1981 | Forio (Napoli) |
| V13-P08 | 4 | A(2); P(1); S(1) | 1961 | Ischia (Napoli) |
| V13-P09 | 19 | A(19) | -- | Portici (Napoli) |
| V13-P10 | 6 | P(2); S(4) | -- | Lapio (Avellino) |
| V13-P11 | 11 | P(9); S(2) | 1964 | Maddaloni (Caserta) |
| V13-P12 | 19 | A(14); P(5) | 1980 | Ischia (Napoli) |
| V13-P13 | 77 | A(47); N(2); P(21); S(7) | 1964 | Maddaloni (Caserta) |
| V13-P14 | 20 | A(1); P(16); S(3) | 1965 | Lapio (Avellino) |
| V13-P15 | 13 | S(13) | 1967 | Portici (Napoli) |
| V13-P16 | 21 | P(20); S(1) | 1964 | Pertosa (Salerno) |
| V13-P17 | 13 | A(6); P(5); S(2) | 1962 | Isola di Vivara (Procida, Napoli) |
| V13-P18 | 10 | A(10) | -- | Portici? (Napoli) |
| V13-P19 | 21 | A(1); P(18); S(2) | 1964 | Sarno (Salerno) |
| V13-P20 | 48 | A(1); P(43); S(4) | 1965 | Portici (Napoli) |
| V13-P21 | 8 | A(1); P(3); S(4) | -- | Portici (Napoli) |
| V13-P22 | 26 | P(12); S(14) | -- | Portici (Napoli)* |

**Table S14.** Termite specimens from Springhetti Collection. Jar 14: Italian termites, *K. flavicollis*. All abbreviations as in Table S1 and S2.

| Test tube code | Number of individuals | Castes | Year of collection | Administrative Region | Municipality/Locality |
|---|---|---|---|---|---|
| V14-P01 | 7 | S(7) | 1966 | Tuscany | Viareggio (Lucca) |
| V14-P02 | 14 | P(13); S(1) | -- | Tuscany | Viareggio (Lucca)* |
| V14-P03 | 57 | P(21); R(2); S(34) | 1966 | Tuscany | Viareggio (Lucca) |
| V14-P04 | 14 | P(9); S(5) | 1966 | Tuscany | Massarosa (Lucca) |
| V14-P05 | 18 | A(1); N(1); P(8); S(8) | 1966 | Tuscany | Viareggio (Lucca) |
| V14-P06 | 1 | R(1) | 1966 | Tuscany | Pisa |
| V14-P07 | 13 | A(13) | -- | Tuscany | Viareggio (Lucca) |
| V14-P08 | 14 | A(14) | -- | Tuscany | Viareggio (Lucca) |
| V14-P09 | 12 | P(10); S(2) | 1976 | Tuscany | Carrara (Massa-Carrara) |
| V14-P10 | 9 | A(3); P(3); S(3) | 1991 | Tuscany | Marina di Alberese (Grosseto) |
| V14-P11 | 27 | A(1); P(13); S(13) | -- | Tuscany | Poggibonsi (Siena) |
| V14-P12 | 45 | P(45) | 1978 | Tuscany | Volterra (Pisa) |
| V14-P13 | 1 | S(1) | 1958 | Marche | Mondolfo (Pesaro e Urbino) |
| V14-P14 | 68 | A(2); P(63); S(3) | -- | Abruzzo | Orsogna (Chieti) |
| V14-P15 | 10 | A(10) | -- | Marche | Ancona |
| V14-P16 | 21 | P(19); S(2) | -- | Abruzzo | Francavilla al Mare (Chieti) |
| V14-P17 | 13 | A(3); P(7); S(3) | -- | Abruzzo | Pescara |
| V14-P18 | 10 | A(10) | -- | Marche | Ancona |
| V14-P19 | 25 | A(3); P(15); S(7) | -- | Abruzzo | Pescara |
| V14-P20 | 29 | P(11); S(18) | -- | Abruzzo | Miglianico (Chieti) |
| V14-P21 | 6 | A(2); P(3); S(1) | 1970 | Abruzzo | Poggiofiorito (Chieti) |
| V14-P22 | 10 | P(8); S(2) | 1973 | Abruzzo | Bucchianico (Chieti) |
| V14-P23 | 24 | A(16); P(8) | -- | Umbria | Amelia (Terni) |
| V14-P24 | 17 | P(4); S(13) | -- | Emilia Romagna | Migliarino (Fiscaglia, Ferrara) |
| V14-P25 | 18 | P(16); S(2) | 1958 | Marche | Grottammare (Ascoli Piceno) |
| V14-P26 | 49 | P(28); S(21) | 1966 | Marche | Ancona |
| V14-P27 | 436 | A(436) | -- | Marche | Ancona |
| V14-P28 | 34 | P(32); S(2) | -- | Marche | Senigallia |
| V14-P29 | 52 | P(47); S(5) | 1973 | Abruzzo | Chieti |
| V14-P30 | 47 | A(4); P(26); S(17) | 1966 | Marche | Ancona |
| V14-P31 | 44 | P(26); S(18) | 1966 | Marche | Ancona |
| V14-P32 | 12 | A(6); P(2); S(4) | 1958 | Marche | Ancona |
| V14-P33 | 32 | A(32) | -- | Marche | Ancona |
| V14-P34 | 18 | A(18) | -- | Marche | Ancona |
| V14-P35 | 22 | P(12); S(10) | -- | Marche | Ancona |
| V14-P36 | 31 | P(21); S(10) | 1966 | Marche | Ancona |
| V14-P37 | 45 | P(34); S(11) | -- | Abruzzo | Miglianico (Chieti) |
| V14-P38 | 16 | P(1); S(15) | 1966 | Abruzzo | Pescara |
| V14-P39 | 22 | A(22) | -- | Marche | Ancona |
| V14-P40 | 14 | A(14) | -- | Abruzzo | Montesilvano (Pescara)* |
| V14-P41 | 62 | P(38); S(24) | 1966 | Marche | Ancona |
| V14-P42 | 41 | A(2); P(35); S(4) | 1973 | Abruzzo | Chieti |
| V14-P43 | 35 | P(20); S(15) | -- | Marche | Ancona |

**Table S15.** Termite specimens from Springhetti Collection. Jar 17, foreign termites: *Mastotermes darwiniensis*, Australia. All abbreviations as in Table S1.

| Test tube code | Number of individuals | Castes | Year of collection | State | Locality |
|---|---|---|---|---|---|
| V17-P01 | 97 | A(6); S(1); W(90) | 1913 | Northern Territory of Australia | Koolpinyah |
| V17-P02 | 4 | A(1); S(2); W(1) | -- | Queensland | -- |
| V17-P03 | 13 | A(13) | 1953 | -- | -- |
| V17-P04 | 5 | W(5) | -- | -- | -- |
| V17-P05 | 802 | S(11); W(791) | 1959 | Queensland | Rita Island |

**Table S16.** Termite specimens from Springhetti Collection. Jar 18, foreign termites: Hodotermitidae from Africa (except V18-P03 from United States). All abbreviations as in Table S1.

| Test tube code | Number of individuals | Castes | Year of collection | State | Locality |
|---|---|---|---|---|---|
| V18-P01 | 25 | W(25) | 1959 | Egypt | Cairo Governorate |
| V18-P02 | 34 | A(24); W(10) | 1960 | Egypt | -- |
| V18-P03 | 50 | N(17); S(1); W(32) | 1981 | California (USA) | -- |
| V18-P04 | 8 | S(4); W(4) | -- | --- | -- |
| V18-P05 | 91 | W(91) | 1960 | Egypt | Ismailia Governorate |
| V18-P06 | 74 | W(74) | 1959 | Egypt | -- |
| V18-P07 | 162 | A(34); W(128) | 1958 | Egypt | Abu Rawash (Cairo Governorate) |
| V18-P08 | 116 | S(5); W(111) | 1960 | Egypt | -- |
| V18-P09 | 310 | W(310) | 1958 | Egypt | Damanhur (Beheira Governorate) |
| V18-P10 | 216 | W(216) | 1959 | Egypt | Hawsh 'Isa (Beheira Governorate) |
| V18-P11 | 129 | A(40); S(1); W(88) | 1960 | Egypt | Tell El Kebir (Ismailia Governorate) |
| V18-P12 | 105 | W(105) | 1959 | Egypt | Cairo Governorate |
| V18-P13 | 71 | A(3); S(3); W(65) | 1960 | Egypt | Abu Hammad (Sharqia Governorate) |
| V18-P14 | 59 | A(6); W(53) | 1960 | Egypt | Abu Hammad (Sharqia Governorate) |

**Table S17.** Termite specimens from Springhetti Collection. Jar 19, foreign termites: unidentified from Africa and Pakistan. All abbreviations as in Table S1.

| Test tube code | Number of individuals | Castes | Year of collection | State | Locality |
|---|---|---|---|---|---|
| V19-P01 | 1 | R(1) | 1952 | Uganda | -- |
| V19-P02 | 1 | R(1) | 1953 | Central African Republic | -- |
| V19-P03 | 2 | 2(2) | 1952 | Uganda | Agoro (Northern Region) |
| V19-P04 | 3 | R(1); W(2) | 1952 | Uganda | Agoro (Northern Region) |
| V19-P05 | 1 | R(1) | -- | -- | -- |
| V19-P06 | 5 | R(1); S(4) | 1952 | Uganda | Agoro (Northern Region) |
| V19-P07 | 1 | R(1) | 1953 | Central African Republic | -- |
| V19-P08 | 8 | R(1); S(4); W(3) | 1952 | Uganda | Agoro (Northern Region) |
| V19-P09 | 7 | R(3); W(4) | 1952 | Uganda | -- |
| V19-P10 | 1 | R(1) | 1952 | Uganda | -- |
| V19-P11 | 70 | A(70) | 1953 | Pakistan | -- |

**Table S18.** Termite specimens from Springhetti Collection. Jar 20, foreign and Italian termites belonging to 5 families from all continents. SE, secondary reproducers. All other abbreviations as in Table 1.

| Test tube code | Taxon | Number of individuals | Castes | Year fo collection | State | Locality |
|---|---|---|---|---|---|---|
| V20-P01 | *Zootermopsis angusticollis* | 1 | SE(1) | 1934 | California | Berkeley |
| V20-P02 | *Zootermopsis angusticollis* | 57 | P(56); SE(1) | -- | California | -- |
| V20-P03 | *Reticulitermes lucifugus* | 5 | A(5) | 1952 | Italy | Genova |
| V20-P04 | *Zootermopsis angusticollis* | 30 | A(29); P(1) | -- | California | -- |
| V20-P05 | *Procryptotermes* sp. | 5 | S(1); W(4) | -- | -- | -- |
| V20-P06 | *Archotermopsis wroughtoni* | 2 | S(1); W(1) | 1915 | India | -- |
| V20-P07 | *Kalotermes minor* | 3 | A(3) | -- | Arizona | Sabino Canyon |
| V20-P08 | *Psammotermes assuanensis* | 36 | A(12); S(15); W(9) | 1959 | Egypt | Ain Shams (Cairo Governorate) |
| V20-P09 | *Nasutitermes* sp. | 18 | A(4); S(8); W(6) | 1971 | -- | -- |
| V20-P10 | Unidentified | 29 | A(3); S(13); W(13) | -- | Pakistan | -- |
| V20-P11 | *Stolotermes africanus* | 3 | S(2); W(1) | 1935 | South Africa | Coldstream (Eastern Cape) |
| V20-P12 | Unidentified | 57 | S(2); W(55) | 1968 | Venezuela | -- |
| V20-P13 | *Zootermopsis angusticollis* | 26 | N(1); P(25) | -- | California | -- |
| V20-P14 | *Zootermopsis angusticollis* | 2 | S(2) | 1934 | California | Berkeley |
| V20-P15 | Unidentified | 17 | S(17) | 1960 | Brazil | -- |
| V20-P16 | *Zootermopsis angusticollis* | 43 | N(8); P(35) | -- | California | -- |
| V20-P17 | Unidentified | 5 | S(5) | -- | Benin | Abomey (Zou Department) |
| V20-P18 | Unidentified | 13 | S(1); W(12) | -- | Benin | Abomey (Zou Department) |
| V20-P19 | *Procryptotermes* sp. | 22 | S(4); W(18) | -- | -- | -- |
| V20-P20 | *Cubitermes gibbifrons* | 34 | S(6); W(28) | 1957 | Democratic Republic of Congo | Yangambi (Oriental Province) |
| V20-P21 | *Zootermopsis angusticollis* | 4 | A(3); W(1) | -- | California | -- |
| V20-P22 | *Kalotermes* sp. | 3 | A(2); W(1) | -- | Texas | Brownsville |
| V20-P23 | *Reticulitermes flavipes* | 65 | A(65) | -- | -- | -- |
| V20-P24 | *Reticulitermes flavipes* | 55 | N(11); W(29) S(15) | -- | Germany | Leverkusen (North Rhine-Westphalia) |
| V20-P25 | *Archotermopsis wroughtoni* | 8 | S(2); W(6) | 1962 | India | Achabal (Kashmir Division) |
| V20-P26 | *Kalotermes shwarzi* | 5 | A(5) | -- | Florida | Paradise Key |
| V20-P27 | *Kalotermes hubbardi* | 4 | A(4) | -- | Arizona | Sabino canyon |
| V20-P28 | *Reticulitermes lucifugus?* | 2 | R(2) | -- | -- | -- |
| V20-P29 | *Stolotermes brunneicornis* | 6 | A(6) | 1878 | New Zealand | Titirangi (Auckland) |
| V20-P30 | *Porotermes planiceps* | 9 | S(8); W(1) | 1938 | South Africa | Wilderness (Western Cape) |
| V20-P31 | *Reticulitermes hesperus* | 11 | A(8); W(3) | 1951 | California | Sacramento |
| V20-P32 | *Psammotermes assuanensis* | 54 | A(19); S(35) | 1959 | Egypt | -- |
| V20-P33 | *Reticulitermes virginicus* | 6 | A(6) | -- | Virginia | Roanoke |
| V20-P34 | *Zootermopsis angusticollis* | 5 | A(5) | -- | State of Washington | Olympia |
| V20-P35 | *Schedorhinotermes javanicus* | 26 | S(12); W(14) | -- | Indonesia | Fort de Kock (West Sumatra) |
| V20-P36 | *Kalotermes flavicollis* | 11 | A(11) | -- | Italy | Apulia (Region) |
| V20-P37 | *Porotermes planiceps* | 15 | A(14);W(1) | 1935 | South Africa | Wilderness (Western Cape) |
| V20-P38 | *Reticulitermes virginicus* | 77 | A(77) | 1949 | South Carolina | Spartanburg |

| | | | | | | |
|---|---|---|---|---|---|---|
| V20-P39 | *Coptotermes sjostedti* | 4 | A(4) | 1948 | Democratic Republic of Congo | -- |
| V20-P40 | *Psammotermes fuscofemoralis?* | 97 | S(11); W(86) | 1958 | Egypt | -- |
| V20-P41 | *Neotermes* sp. | 23 | A(2); S(1); W(20) | -- | -- | Rio Tanaro (?) |
| V20-P42 | *Coptotermes lacteus* | 72 | S(33); W(39) | -- | -- | -- |
| V20-P43 | *Reticulitermes tibialis?* | 32 | A(27); S(1); W(4) | -- | -- | -- |
| V20-P44 | *Kalotermes flavicollis* | 62 | S(62) | 1954 | Italy | Apulia (Region) |
| V20-P45 | *Coptotermes lacteus* | 39 | S(11); W(28) | -- | -- | -- |

**Table S19.** Termite specimens from Springhetti Collection. Jar 21, foreign termites from Africa, Asia, America and Oceania. The species are mostly unidentified. All abbreviations as in Table S1.

| Test tube code | Taxon | Number of individuals | Castes | Year of collection | State | Locality |
|---|---|---|---|---|---|---|
| V21-P01 | Unidentified | 1 | R(1) | 1953 | Senegal | -- |
| V21-P02 | *Bellicositermes jeanneli?* | 15 | S(15) | 1952 | Uganda | -- |
| V21-P03 | Unidentified | 1 | R(1) | 1953 | Filippine | Manila |
| V21-P04 | Unidentified | 1 | R(1) | 1960 | Somalia? | -- |
| V21-P05 | Unidentified | 11 | A(11) | 1952 | Uganda | -- |
| V21-P06 | Unidentified | 74 | S(56); W(18) | 1953 | Central African Republic | -- |
| V21-P07 | Unidentified | 185 | R(1); S(31); W(153) | 1953 | Senegal | -- |
| V21-P08 | Unidentified | 239 | A(3); S(64); W(172) | 1953 | Central African Republic | -- |
| V21-P09 | Unidentified | 554 | S(36); W(510); A(7); R(1) | 1953 | Central African Republic | -- |
| V21-P10 | Unidentified | 132 | R(1); S(108); W(23) | 1952 | Uganda | Agoro (Northern Region) |
| V21-P11 | Unidentified | 456 | S(86); W(370) | 1953 | Filippine | Manila |
| V21-P12 | *Psammotermes assuanensis* | 307 | W(307) | 1959 | Egypt | -- |
| V21-P13 | Unidentified | 369 | S(11); W(358) | 1956 | New Guinea island | -- |
| V21-P14 | Unidentified | 1135 | W(822); S(313) | 1964 | Panama | El Chorrillo |
| V21-P15 | Unidentified | 533 | W(363); S(170) | 1953 | Pakistan | -- |

**Table S20.** Termite specimens from Springhetti Collection. Jar 22, foreign termites from Africa, Asia and America. The species are mostly unidentified. All abbreviations as in Table S1.

| Test tube code | Taxon | Numbe of individuals | Castes | Year of collection | State | Locality |
|---|---|---|---|---|---|---|
| V22-P01 | *Odontotermes badius* | 23 | A(23) | 1952 | Kenya | Muguga (Kiambu County) |
| V22-P02 | Unidentified | 48 | S(31); W(17) | 1960 | Brazil | -- |
| V22-P03 | Unidentified | 35 | N(2); R(1); W(32) | 1960 | Brazil | -- |
| V22-P04 | Unidentified | 18 | S(3); W(15) | 1960 | Brazil | -- |
| V22-P05 | Unidentified | 10 | S(10) | 1960 | Brazil | -- |
| V22 -P06 | Unidentified | 1 | R(1) | 1953 | Senegal | -- |
| V22-P07 | *Reticulitermes lucifugus?* | 15 | A(15) | -- | -- | -- |
| V22-P08 | Unidentified | 11 | N(10); R(1) | 1952 | Sierra Leone | -- |
| V22-P09 | Unidentified | 164 | A(56); S(104); W(4) | 1960 | Egypt | -- |
| V22-P10 | Unidentified | 56 | S(37); W(19) | 1960 | Brazil | -- |
| V22-P11 | Unidentified | 83 | R(2); S(52); W(29) | 1960 | Somalia? | -- |
| V22-P12 | Unidentified | 78 | A(2); W(76) | 1960 | Egypt | -- |
| V22-P13 | Unidentified | 664 | S(584); W(80) | 1960 | Senegal | Bambey (Diourbel Region) |
| V22-P14 | *Schedorhinotermes javanicus* | 22 | A(22) | 1934 | Sumatra | Fort de Kock (West Sumatra) |
| V22-P15 | Unidentified | 425 | A(1); S(154); W(270) | 1960 | Senegal | Bambey (Diourbel Region) |
| V22-P16 | *Eutermes* sp. | 1158 | S(336); W(822) | 1924 | Sumatra | -- |
| V22-P17 | Unidentified | 431 | S(390); W(41) | 1960 | Senegal | Bambey (Diourbel Region) |
| V22-P18 | Unidentified | 604 | S(526); W(78) | 1954 | Filippine | Manila |

**Table S21.** Termite specimens from Springhetti Collection. Jar 23, foreign termites from Africa, Asia and America. The species are mostly unidentified. All abbreviations as in Table S1.

| Test tube code | Taxon | Number of individuals | Castes | Year of collection | State | Locality |
|---|---|---|---|---|---|---|
| V23-P01 | Unidentified | 1 | R(1) | 1953 | Central African Republic | -- |
| V23-P02 | *Pseudacanthotermes minor?* | 26 | A(3); S(5); W(18) | -- | Uganda | -- |
| V23-P03 | Unidentified | 34 | A(9); N(6); S(7); W(12) | 1955 | Japan | -- |
| V23-P04 | Unidentified | 198 | N(4); W(182); S(12) | 1955 | Japan | -- |
| V23-P05 | Unidentified | 69 | A(26); R(2); S(30); W(11) | 1953 | Kenya | -- |
| V23-P06 | Unidentified | 52 | S(32); W(20) | 1954 | Mozambico | Zambezia Province |
| V23-P07 | Unidentified | 41 | A(13); S(28) | 1954 | Central African Republic | -- |
| V23-P08 | Unidentified | 88 | S(38); W(50) | 1952 | Central African Republic | -- |
| V23-P09 | Unidentified | 49 | A(48); S(1) | 1953 | Central African Republic | -- |
| V23-P10 | Unidentified | 81 | R(1); S(22); W(58) | 1952 | Uganda | -- |
| V23-P11 | Unidentified | 569 | S(57); W(512) | 1955 | Argentina | Formosa (Formosa Department) |
| V23-P12 | Unidentified | 53 | S(39); W(14) | 1952 | Uganda | -- |
| V23-P13 | Unidentified | 1 | S(1) | 1952 | Sierra Leone | -- |
| V23-P14 | Unidentified | 84 | S(24); W(60) | 1952 | Tanzania | -- |
| V23-P15 | Unidentified | 74 | A(33); S(41) | 1953 | Central African Republic | -- |
| V23-P16 | Unidentified | 467 | N(1); R(2); S(38); W(426) | 1953 | Kenya | -- |
| V23-P17 | *Schedorhinotermes javanicus* | 8 | A(8) | 1922 | Sumatra | Fort de Kock (West Sumatra) |
| V23-P18 | Unidentified | 7 | N(2); S(2); W(3) | 1955 | Japan | Tokyo |
| V23-P19 | Unidentified | 57 | S(55); W(2) | 1954 | Mozambico | Zambezia Province |
| V23-P20 | Unidentified | 546 | N(7); W(539) | 1952 | Sierra Leone | -- |
| V23-P21 | Unidentified | 95 | S(52); W(43) | 1953 | Kenya | -- |
| V23-P22 | *Schedorhinotermes sp.* | 76 | S(54); W(22) | -- | -- | -- |
| V23-P23 | Unidentified | 113 | S(2); W(111) | 1953 | Kenya | -- |
| V23-P24 | Unidentified | 96 | S(1); W(95) | 1952 | Uganda | -- |
| V23-P25 | Unidentified | 133 | W(133) | 1954 | Mozambico | Zambezia Province |
| V23-P26 | Unidentified | 724 | N(2); S(55); W(667) | 1955 | Argentina | Formosa (Formosa Department) |
| V23-P27 | Unidentified | 444 | S(27); W(417) | 1952 | Sierra Leone | -- |

**Table S22.** Termite specimens from Springhetti Collection. Jar 24, foreign termites from Africa, Asia and Oceania. The species are mostly unidentified. All abbreviations as in Table S1.

| Test tube code | Taxon | Number of individuals | Castes | Year of collection | State | Locality |
|---|---|---|---|---|---|---|
| V24-P01 | Unidentified | 1 | R(1) | 1964 | Somalia? | -- |
| V24-P02 | Unidentified | 198 | S(110); W(88) | 1964 | Somalia? | -- |
| V24-P03 | Unidentified | 10 | A(10) | 1964 | Somalia? | -- |
| V24-P04 | Unidentified | 88 | A(2); S(19); W(67) | 1952 | Uganda | -- |
| V24-P05 | Unidentified | 35 | S(35) | 1952 | Uganda | -- |
| V24-P06 | Unidentified | 66 | S(56); W(10) | 1952 | Sierra Leone | -- |
| V24-P07 | Unidentified | 50 | S(50) | 1952 | Sierra Leone | -- |
| V24-P08 | Unidentified | 18 | W(18) | 1953 | Filippine | Manila |
| V24-P09 | Unidentified | 9 | S(9) | 1952 | Central African Republic | -- |
| V24-P10 | Unidentified | 227 | N(5); S(18); W(204) | 1956 | New Guinea island | -- |
| V24-P11 | Unidentified | 750 | A(227); S(29); W(494) | 1952 | Sierra Leone | -- |
| V24-P12 | Unidentified | 228 | W(228) | 1960 | Somalia? | -- |
| V24-P13 | Unidentified | 326 | S(35); W(291) | 1952 | Central African Republic | -- |
| V24-P14 | *Psammotermes fuscofemoralis* | 844 | W(844) | 1958 | Egypt | Luxor |
| V24-P15 | Unidentified | 466 | S(30); W(436) | 1971 | Sri Lanka | Sigiriya (Central Province) |

**Table S23.** Termite specimens from Springhetti Collection. Jar 26, foreign termites from Africa, unidentified. Abbreviation as in Table S1.

| Test tube code | Number of individuals | Caste | Year of collection | State | Locality |
|---|---|---|---|---|---|
| V26-P01 | 136 | A(136) | 1952 | Uganda | Agoro (Northern Region) |

**Table S24.** Termite specimens from Springhetti Collection. Jar 29, foreign termites from Africa, America and Asia. The species are mostly unidentified. All abbreviations as in Table S1.

| Test tube code | Taxon | Number of individuals | Castes | Year of collection | State | Locality |
|---|---|---|---|---|---|---|
| V29-P01 | Unidentified | 14 | S(4); W(10) | -- | Egypt | Imbaba (Giza Governorate) |
| V29-P02 | Unidentified | 70 | S(70) | 1952 | Uganda | -- |
| V29-P03 | Unidentified | 101 | S(3); W(98) | 1958 | Brazil | -- |
| V29-P04 | Unidentified | 126 | S(82); W(44) | -- | Bahama | -- |
| V29-P05 | Unidentified | 239 | S(222); W(17) | 1954 | Filippine | Manila |
| V29 -P06 | *Cornitermes cumulans* | 702 | N(37); S(201); W(464) | 1958 | Brazil | São Paulo |
| V29-P07 | Unidentified | 80 | N(14); S(3); W(63) | 1953 | Brazil | -- |

**Table S25.** Termite specimens from Springhetti Collection. Jar 30, foreign termites (mostly of the family Termitidae) from Africa, Asia, America and Oceania. All abbreviations as in Table S1.

| Test tube code | Taxon | Number of individuals | Castes | Year of collection | State | Locality |
|---|---|---|---|---|---|---|
| V30-P01 | Unidentified | 9 | A(9) | 1953 | Uganda | Jinja (Eastern Region) |
| V30-P02 | Unidentified | 12 | A(4); S(3); W(5) | 1963? | Australia | -- |
| V30-P03 | Unidentified | 78 | A(9); S(29); W(40) | 1953 | Pakistan | -- |
| V30-P04 | *Bellicositermes bellicosus?* | 13 | S(10); W(3) | 1953 | Senegal | -- |
| V30-P05 | Unidentified | 15 | S(6); W(9) | 1953 | Kenya | -- |
| V30-P06 | Unidentified | 1 | R(1) | 1957 | Brazil | -- |
| V30-P07 | *Bellicositermes goliath?* | 13 | A(3); S(5); W(5) | 1953 | Central African Republic | -- |
| V30-P08 | *Bellicositermes goliath?* | 13 | S(9); W(4) | 1953 | Central African Republic | -- |
| V30-P09 | *Bellicositermes natalensis?* | 25 | S(25) | 1952 | Sierra Leone | -- |
| V30-P10 | Unidentified | 1 | R(1) | 1953 | Central African Republic | -- |
| V30-P11 | *Cornitermes cumulans* | 1 | R(1) | 1957 | Brazil | -- |
| V30-P12 | *Bellicositermes jeanneli?* | 16 | S(13); W(3) | 1952 | Uganda | -- |
| V30-P13 | *Odontotermes tanganicus?* | 11 | S(6); W(5) | 1952 | Tanzania | -- |
| V30-P14 | *Bellicositermes ukuzii* | 12 | S(10); W(2) | 1954 | Mozambico | Zambezia Province |
| V30-P15 | Unidentified | 14 | S(7); W(7) | 1972 | Etiopia | Hawassa (Sidama Region) |
| V30-P16 | *Bellicositermes natalensis?* | 35 | S(5); W(30) | 1952 | Uganda | -- |
| V30-P17 | Unidentified | 50 | S(6); W(44) | 1953 | Filippine | Manila |
| V30-P18 | Unidentified | 37 | S(2); W(35) | 1952 | Central African Republic | -- |
| V30-P19 | Unidentified | 70 | N(2); S(14); W(54) | 1952 | Sierra Leone | -- |
| V30-P20 | Unidentified | 287 | A(1); N(276); S(2); W(8) | 1955 | Argentina | Formosa (Formosa Department) |
| V30-P21 | Unidentified | 16 | S(16) | 1954 | Filippine | Manila |
| V30-P22 | Unidentified | 12 | S(5); W(7) | | -- | -- |
| V30-P23 | Unidentified | 37 | W(37) | 1954 | Japan | Tokyo |
| V30-P24 | Unidentified | 96 | S(87); W(9) | 1965 | Panama | Parque Lefevre |
| V30-P25 | Unidentified | 20 | S(13); W(7) | 1964 | Panama | -- |
| V30-P26 | *Reticulitermes lucifugus?* | 68 | A(28); N(15); S(12); W(13) | -- | -- | -- |
| V30-P27 | Unidentified | 52 | N(16); S(12); W(24) | 1952 | Sierra Leone | -- |
| V30-P28 | Unidentified | 85 | S(5); W(80) | 1955 | Argentina | Formosa (Formosa Department) |
| V30-P29 | Unidentified | 55 | N(3); S(12); W(40) | 1955 | Argentina | -- |
| V30-P30 | *Odontotermes tanganicus?* | 68 | A(4); S(4); W(60) | 1953 | Kenya | -- |

**Table S26.** Termite specimens from Springhetti Collection. Jar 31, foreign termites from Africa and Asia. All abbreviations as in Table S1.

| Test tube code | Taxon | Number of individuals | Castes | Year of collection | State | Locality |
|---|---|---|---|---|---|---|
| V31-P01 | Unidentified | 215 | S(109); W(106) | 1959 | Senegal | Bambey (Diourbel Region) |
| V31-P02 | Unidentified | 123 | A(17); S(34);W(72) | 1952 | Uganda | Jinja (Eastern Region) |
| V31-P03 | Unidentified | 90 | A(90) | 1952 | Uganda | Jinja (Eastern Region) |
| V31-P04 | *Anacanthotermes ochraceus* | 32 | W(32) | 1959 | Egypt | Zagazig (Sharqia Governorate) |
| V31-P05 | Unidentified | 42 | S(31); W(11) | 1959 | Senegal | Bambey (Diourbel Region) |
| V31-P06 | Unidentified | 261 | R(2); S(95); W(164) | 1959 | Senegal | Bambey (Diourbel Region) |
| V31-P07 | Unidentified | 121 | N(14); S(11); W(96) | 1952 | Zambia | -- |
| V31-P08 | *Amitermes desertorum?* | 390 | A(160); W(230) | 1958 | Egypt | Mansoura (Dakahlia Governorate) |
| V31-P09 | Unidentified | 144 | S(96); W(48) | 1959 | Senegal | Bambey (Diourbel Region) |
| V31-P10 | Unidentified | 157 | S(89); W(68) | 1960 | Senegal | Bambey (Diourbel Region) |
| V31-P11 | *Psammotermes assuanensis* | 257 | A(90); W(167) | 1959 | Egypt | Ain Shams (Cairo Governorate) |
| V31-P12 | Unidentified | 223 | A(39); N(14); W(170) | 1952 | Malaysa | Sandakan (Sandakan Division) |
| V31-P13 | Unidentified | 117 | S(65); W(52) | 1959 | Senegal | Bambey (Diourbel Region) |
| V31-P14 | *Amitermes desertorum?* | 242 | A(164); W(78) | 1959 | Egypt | Mansoura (Dakahlia Governorate) |
| V31-P15 | *Amitermes desertorum?* | 273 | N(3); W(270) | 1958 | Egypt | Mansoura (Dakahlia Governorate) |

**Table S27.** Termite specimens from Springhetti Collection. Jar 32, foreign termites from Africa, Asia, America and Europe. All abbreviations as in Table S1.

| Test tube code | Taxon | Number of individuals | Castes | Year of collection | State | Locality |
|---|---|---|---|---|---|---|
| V32-P01 | *Thoracotermes macrothorax* | 30 | N(5); S(5); W(20) | 1957 | Democratic Republic of the Congo | Yangambi-Kisangani |
| V32-P02 | Unidentified | 62 | N(2); S(26); W(34) | 1960 | Brazil | -- |
| V32-P03 | *Ophiotermes mirandus* | 31 | S(1); W(30) | 1957 | Democratic Republic of the Congo | Yangambi-Kisangani |
| V32-P04 | *Apilitermes longiceps* | 33 | S(5); W(28) | 1957 | Democratic Republic of the Congo | Yangambi-Kisangani |
| V32-P05 | *Bellicositermes convexus?* | 8 | S(8) | 1952 | Sierra Leone | -- |
| V32-P06 | *Eutermes aethiops* | 32 | S(15); W(17) | 1957 | Democratic Republic of the Congo | Yangambi-Kisangani |
| V32-P07 | *Eutermes incurvus* | 30 | S(12); W(18) | 1957 | Democratic Republic of the Congo | Yangambi-Kisangani |
| V32-P08 | *Cubitermes gibbifrons* | 25 | A(1); S(7); W(17) | 1958 | Democratic Republic of the Congo | Yangambi-Kisangani |
| V32-P09 | Unidentified | 4 | A(4) | 1922 | Sumatra | Fort de Kock |
| V32-P10 | *Archotermopsis wroughtoni* | 2 | W(2) | -- | India | -- |
| V32-P11 | Unidentified | 21 | S(13); W(8) | 1960 | Brazil | -- |
| V32-P12 | Unidentified | 34 | S(22); W(12) | 1961 | Brazil | -- |
| V32-P13 | Unidentified | 36 | P(35); S(1) | 1960 | Spain | Murcia |
| V32-P14 | Unidentified | 15 | S(2); W(13) | 1954 | Filippine | Manila |
| V32-P15 | Unidentified | 51 | S(49); W(2) | 1960 | Brazil | -- |
| V32-P16 | Unidentified | 9 | A(9) | 1953 | Central African Republic | -- |
| V32-P17 | *Pericapritermes heteronotus* | 25 | S(10); W(15) | 1957 | Democratic Republic of the Congo | Yangambi-Kisangani |
| V32-P18 | Unidentified | 65 | N(2); W(63) | 1961 | Costa Rica | -- |
| V32-P19 | Unidentified | 182 | S(182) | 1961 | Costa Rica | -- |

**Table S28.** Termite specimens from Springhetti Collection. Jar 33, foreign termites (mostly of the family Termitidae) from Africa, Asia and America. All abbreviations as in Table S1.

| Test tube code | Taxon | Number of individuals | Castes | Year of collection | State | Locality |
|---|---|---|---|---|---|---|
| V33-P01 | *Macrotermes natalensis* | 5 | A(5) | 1948 | Democratic Republic of the Congo | -- |
| V33-P02 | *Syntermes spinosus* | 27 | N(1); S(6); W(20) | 1920 | Guyana | Kartabo (Cuyuni-Mazaruni) |
| V33-P03 | *Thoracotermes macrothorax* | 5 | S(4); W(1) | -- | -- | -- |
| V33-P04 | *Macrotermes natalensis?* | 17 | S(15); W(2) | -- | -- | -- |
| V33-P05 | Unidentified | 10 | A(8); N(2) | 1953 | Argentina | Tucumán Province |
| V33-P06 | *Microtermes fuscofemoralis* | 7 | S(6); W(1) | -- | -- | -- |
| V33-P07 | *Acanthotermes acanthothorax* | 17 | S(12); W(5) | -- | -- | -- |
| V33-P08 | Unidentified | 52 | S(3); W(49) | 1981 | USA | California |
| V33-P09 | *Amitermes desertorum?* | 31 | A(8); S(1); W(22) | 1959 | Egypt | Mansura (Dakahlia Governorate) |
| V33-P10 | Unidentified | 19 | A(6); N(2); W(11) | 1953 | Argentina | Tucumán Province |
| V33-P11 | Unidentified | -- | Eggs | 1953 | Argentina | Tucumán Province |
| V33-P12 | *Capritermes padangensis* | 9 | S(3); W(6) | 1922 | Sumatra | Fort de Kock (West Sumatra) |
| V33-P13 | Unidentified | 27 | W(27) | 1953 | Argentina | Tucumán Province |
| V33-P14 | Unidentified | 10 | A(4); W(6) | 1953 | Argentina | Tucumán Province |
| V33-P15 | Unidentified | 30 | W(30) | 1953 | Argentina | Tucumán Province |
| V33-P16 | Unidentified | -- | Eggs | -- | -- | -- |
| V33-P17 | Unidentified | 11 | S(4); W(7) | -- | Sierra Leone | -- |
| V33-P18 | Unidentified | 31 | S(15); W(16) | 1981 | USA | Berkeley (California) |
| V33-P19 | *Acanthotermes desertorum?* | 28 | A(11); S(4); W(13) | 1959 | Egypt | -- |
| V33-P20 | Unidentified | 38 | S(23); W(15) | 1953 | Argentina | Tucumán Province |
| V33-P21 | Unidentified | 112 | S(60); W(52) | 1953 | Argentina | Tucumán Province |
| V33-P22 | *Amitermes desertorum?* | 36 | A(17); S(3); W(16) | 1959 | Egypt | -- |
| V33-P23 | *Nasutitermes* sp | 130 | A(2); N(3); S(3); W(122) | 1953 | Argentina | Tucumán Province |
| V33-P24 | Unidentified | 83 | N(11); W(72) | 1953 | Argentina | Tucumán Province |
| V33-P25 | *Nasutitermes* sp | 68 | S(18); W(50) | 1953 | Argentina | Tucumán Province |
| V33-P26 | *Nasutitermes* sp. | 16 | S(16) | 1953 | Argentina | Tucumán Province |
| V33-P27 | Unidentified | 13 | S(11); W(2) | -- | -- | -- |
| V33-P28 | Unidentified | 257 | S(77); W(180) | 1961 | Costa Rica | -- |
| V33-P29 | *Nasutitermes* sp. | 50 | S(28); W(22) | 1953 | Argentina | Tucumán Province |

**Table S29.** Termite specimens from Springhetti Collection. Jar 34, foreign termites (mostly of the family Termitidae) from Africa, Asia and Oceania. All abbreviations as in Table S1.

| Test tube code | Taxon | Number of individuals | Castes | Year of collection | State | Locality |
|---|---|---|---|---|---|---|
| V34-P01 | Unidentified | 16 | S(15); W(1) | 1954 | Mozambique | Zambezia Province |
| V34-P02 | Unidentified | 43 | S(39); W(4) | 1956 | Filippine | -- |
| V34-P03 | Unidentified | 354 | S(354) | 1952 | Uganda | -- |
| V34-P04 | Unidentified | 72 | S(72) | 1952 | Sierra Leone | -- |
| V34-P05 | Unidentified | 177 | S(51); W(126) | 1956 | New Guinea island | -- |
| V34-P06 | Unidentified | 130 | S(71); W(59) | 1952 | Sierra Leone | -- |
| V34-P07 | Unidentified | 181 | A(181) | 1952 | Central African Republic | -- |
| V34-P08 | Unidentified | 203 | A(7); S(1); W(195) | 1956 | New Guinea island | -- |
| V34-P09 | Unidentified | 199 | S(31); W(168) | 1956 | New Guinea island | -- |
| V34-P10 | Unidentified | 188 | S(63); W(125) | -- | -- | -- |

**Table S30.** Termite specimens from Springhetti Collection. Jar 37, foreign termites (belonging to 6 families) from all continents. All abbreviations as in Table S1.

| Test tube code | Taxon | Number of individuals | Castes | Year of collection | State | Locality |
|---|---|---|---|---|---|---|
| V37-P01 | Unidentified | 1 | R(1) | 1952 | Uganda | -- |
| V37-P02 | Unidentified | 5 | R(1); S(4) | 1952 | Uganda | -- |
| V37-P03 | Unidentified | 2 | R(2) | 1952 | Uganda | -- |
| V37-P04 | *Mastotermes darwiniensis?* | 1 | A(1) | -- | -- | -- |
| V37-P05 | *Bellicositermes natalensis* | 7 | S(7) | -- | Central African Republic | -- |
| V37-P06 | Unidentified | 23 | A(7); S(5); W(11) | 1952 | Sierra Leone | -- |
| V37-P07 | *Mastotermes darwiniensis* | 14 | N(14) | 1953 | Australia | -- |
| V37-P08 | *Zootermopsis angusticollis* | 5 | S(3); W(2) | 1962 | -- | -- |
| V37-P09 | *Zootermopsis nevadensis* | 6 | A(5); S(1) | 1937 | USA | Camino (California) |
| V37-P10 | Unidentified | 4 | R(4) | 1952 | Zimbabwe | -- |
| V37-P11 | *Mastotermes darwiniensis* | 41 | W(41) | -- | Australia | -- |
| V37-P12 | *Odontotermes culturarum* | 16 | S(5); W(11) | 1953 | Kenya | -- |
| V37-P13 | *Anacanthotermes ochraceus* | 13 | S(4); W(9) | 1959 | Egypt | Cairo Governorate |
| V37-P14 | *Anacanthotermes ochraceus* | 34 | S(4); W(30) | 1959 | Egypt | Hawsh Isa (Beheira Governorate) |
| V37-P15 | *Anacanthotermes ochraceus* | 21 | S(3); W(18) | 1959 | Egypt | Zagazig (Sharqia Governorate) |
| V37-P16 | *Anacanthotermes ochraceus* | 21 | A(10); S(1); W(10) | 1959 | Egypt | Abu-Rawash (Cairo Governorate) |
| V37-P17 | *Lacessitermes nemorosus* | 28 | S(9); W(19) | -- | Sumatra | -- |
| V37-P18 | *Anacanthotermes ochraceus?* | 32 | A(1); W(31) | 1958 | Egypt | Damanhur (Beheira Governorate) |
| V37-P19 | *Mastotermes darwiniensis* | 5 | S(5) | 1953 | Australia | -- |
| V37-P20 | Unidentified | 13 | R(13) | 1952 | Sierra Leone | -- |
| V37-P21 | *Lacessitermes nemorosus* | 142 | S(44); W(98) | -- | Sumatra | -- |
| V37-P22 | *Cryptotermes brevis* | 10 | A(7); W(3) | 1952 | Portugal | Madera |
| V37-P23 | *Macrotermes natalensis* | 1 | A(1) | 1948 | Democratic Republic of the Congo | Niangara (Haut-Uélé) |
| V37-P24 | *Lacessititermes jacobsoni* | 24 | R(1); S(11); W(12) | -- | Sumatra | -- |
| V37-P25 | *Capritermes padongensis* | 47 | S(1); W(46) | 1930 | Sumatra | Fort de Kock (West Sumatra) |
| V37-P26 | *Macrotermes muelleri* | 3 | S(3) | -- | Ghana | -- |
| V37-P27 | *Cubitermes bilobatus* | 5 | A(5) | 1959 | Tanzania | -- |
| V37-P28 | Unidentified | 5 | A(5) | -- | -- | -- |
| V37-P29 | *Cubitermes tanganicus?* | 5 | A(5) | 1959 | Tanzania | Ifakara (Kilombero District) |
| V37-P30 | *Coactotermes coarctatus?* | 5 | S(5) | -- | Somalia | Lower Juba (Jubaland) |
| V37-P31 | *Lacessititermes jacobsoni* | 9 | S(5); W(4) | -- | Sumatra | -- |
| V37-P32 | Unidentified | 70 | S(9); W(61) | 1953 | Argentina | Tucumán Province |
| V37-P33 | *Schedorhinotermes javanicus* | 162 | S(35); W(127) | -- | Sumatra | Fort de Kock (West Sumatra) |
| V37-P34 | Unidentified | 39 | W(39) | -- | -- | -- |

**Table S31.** Termite specimens from Springhetti Collection. Jar 38, foreign termites (unidentified) from Africa. All abbreviations as in Table S1.

| Test tube code | Number of individuals | Caste | Year of collection | State | Locality |
|---|---|---|---|---|---|
| V38-P01 | 35 | S(2); W(33) | 1989 | Somalia | Mogadiscio (Banaadir) |
| V38-P02 | 11 | S(4); W(7) | 1989 | Somalia | Mogadiscio (Banaadir) |
| V38-P03 | 93 | A(13); N(2); S(3); W(75) | 1989 | Somalia | Mogadiscio (Banaadir) |
| V38-P04 | 66 | A(10); S(1); W(55) | 1989 | Somalia? | -- |
| V38-P05 | 3 | A(3) | 1989 | Somalia | Mogadiscio (Banaadir) |
| V38-P06 | 10 | S(4); W(6) | -- | -- | -- |
| V38-P07 | 19 | S(5); W(14) | 1989 | Somalia | -- |
| V38-P08 | 51 | S(3); W(48) | 1989 | Somalia | -- |
| V38-P09 | 102 | S(7); W(95) | 1989 | Somalia | -- |
| V38-P10 | 125 | A(1); S(8); W(116) | 1989 | Somalia | -- |
| V38-P11 | 39 | S(13); W(26) | 1989 | Somalia | -- |
| V38-P12 | 92 | S(9); W(83) | 1989 | Somalia | -- |
| V38-P13 | 14 | A(14) | 1989 | Somalia | Mogadiscio (Banaadir) |
